# Exploring the Chemical Profile and Biological Activities of *Eryngium dichotomum*: UHPLC–MS/NMR Characterization, and In Vitro Antioxidant Activity Along with the Antitumor Effect of Falcarinol

**DOI:** 10.3390/molecules31111959

**Published:** 2026-06-04

**Authors:** Roufia Mezaache, Habiba Laraoui, Anis Bertella, Verónica Bastos, Helena Oliveira, Patrick Pale, Aurelien Blanc, Stefan Chassaing, Oana-Crina Bujor, Diana C. G. A. Pinto, Liliana Bădulescu, Artur M. S. Silva, Fatma Bitam

**Affiliations:** 1 Department of Pharmacy, Faculty of Medicine, University Batna 2, Batna 05000, Algeria; r.mezaache@univ-batna2.dz; 2 Laboratory of Chemistry and Environmental Chemistry (L.C.C.E), Department of Chemistry, Faculty of Matter Sciences, University of Batna 1, Batna 05000, Algeria; habiba.laraoui@univ-biskra.dz; 3 Department of Matter Sciences, Faculty of Exact Sciences, University of Biskra, Biskra 07000, Algeria; 4 Laboratory of Applied Molecular Biology (LAMB), Faculty of Life and Nature Sciences, Abbes Laghrour University Khenchela, BP 1252 Road of Batna, Khenchela 40004, Algeria; 5 Food Science Laboratory (LSA), Department of Food Engineering, Institute of Veterinary and Agricultural, Sciences, University Batna 1-Hadj Lakhdar, Batna 05000, Algeria; 6 Department of Biology & CESAM, University of Aveiro, 3810-193 Aveiro, Portugal; veronicabastos@ua.pt (V.B.); holiveira@ua.pt (H.O.); 7 Laboratory of Organometallic Catalysis, Organic Synthesis and Health (COSyS), Institut de Chimie UMR 7177, University of Strasbourg, 67081 Strasbourg, France; ppale@unistra.fr (P.P.); ablanc@unistra.fr (A.B.); chassaing@unistra.fr (S.C.); 8 Research Center for Studies of Food and Agricultural Products Quality, University of Agronomic Sciences and Veterinary Medicine of Bucharest, 59, Mărăști Blvd., 011464 Bucharest, Romania; oana.bujor@qlab.usamv.ro (O.-C.B.); liliana.badulescu@qlab.usamv.ro (L.B.); 9 LAQV-REQUIMTE, Department of Chemistry, University of Aveiro, Campus de Santiago, 3810-193 Aveiro, Portugal; diana@ua.pt (D.C.G.A.P.); artur.silva@ua.pt (A.M.S.S.)

**Keywords:** *Eryngium*, UHPLC–MS, flavonoids, triterpenoid saponins, falcarinol

## Abstract

Qualitative liquid chromatography–mass spectrometry (UHPLC–MS) and NMR analysis of the diethyl ether extract of the aerial part of *Eryngium dichotomum* plant belonging to the Apiaceae family led to the putative identification of phenolic acids, flavonoid glycosides, triterpenoid saponins, fatty acids, and oxylipins. The tentative identification of several secondary metabolites by UHPLC–MS analysis was further confirmed by compound isolation and comprehensive spectroscopic characterization using 2D NMR and mass spectrometry, leading to the elucidation of seven compounds, a mixture of two hydroxy fatty acids, namely (*Z*,*E*)-13-hydroxyoctadeca-9,11-dienoic acid (**1**) and (*E*)-13-hydroxyoctadec-11-enoic acid (**2**); two C17 polyacetylenes, (*E*)-heptadeca-1,10-dien-4,6-diyne-3,8,9-triol (**3**), and falcarinol ((Z)-1,9-heptadeca-1,9-dien-4,6-diyn-3-ol) (**4**); glycerol monopalmitate (**5**) and two flavonoid glycosides, kaempferol 3-*O*-β-D-glucopyranosyl-(1 → 6)-*O*-β-D-galactopyranoside (**6**), and quercetin 3-*O*-β-D-glucopyranosyl-(1 → 6)-*O*-β-D-galactopyranoside (**7**). Furthermore, the antioxidant activity of the *n*-butanol and the diethyl ether extracts of the species were evaluated using the DPPH, FRAP, and ABTS assays. In addition, the anticancer activity of the major falcarinol-type polyacetylene was assessed against A375 human melanoma cells.

## 1. Introduction

The genus *Eryngium* L. (Apiaceae, subfamily Saniculoideae) comprises approximately 300 species distributed worldwide [1]. Numerous *Eryngium* taxa have been used in traditional medicine and are known to produce structurally diverse secondary metabolites, including phenolic acids, flavonoids, coumarins, polyacetylenes, essential oils, and triterpenoid saponins [2]. Among these natural products, polyacetylenes, particularly those of falcarinol type, have been widely reported, and have attracted considerable attention due to their cytotoxic, antimicrobial, anti-inflammatory, and antioxidant activities [3,4,5].

*Eryngium dichotomum* is a Mediterranean plant that is widely distributed in Algeria along with other *Eryngium* species: *E. barrelieri*, *E. triquetrum*, *E. glomeratum*, *E. campestre*, *E. tricuspidatum*, *E. maritimum* and *E. ilicifolium* [6]. To date, only three of these species have been investigated for their phytochemical content, revealing also the presence of polyacetylenes among the major identified secondary metabolites [7,8,9,10]. In contrast, and to the best of our knowledge, very few studies on the phytochemical and biological activities of *E. dichotomum* have been reported. Regarding phytochemical content, investigations of the aerial parts of Tunisian *E. dichotomum* have led to the isolation of stigmasterol and its 3-*O*-β-D-glucoside [11], a glycosylated flavanone (naringenin 7-*O*-α-L-rhamnopyranosyl-(1 → 2)-*O*-β-D-glucopyranoside), and an unusual glucosylated cyclohexenone, as well as a series of sugars (β-D-fructofuranose, 2-*O*-methyl-α-D-fructofuranose, sucrose and D-mannitol) [12]. In a study on all Tunisian *Eryngium* species looking at the variability of antimicrobial and cytotoxicity upon their chemical content, Landoulsi et al. mentioned some antimicrobial activity of *E. dichotomum*, moderate for the aerial parts and stronger for roots, as well as interesting cyctotoxicity against murine tumor cell line [13].

Such a paucity of information on *E. dichotomum* motivated us to engage in a detailed LC–MS phytochemical profiling of *Eryngium dichotomum* grown in Algeria, and to compare the identified metabolite classes and individual compounds with those previously reported in the literature for other *Eryngium* species [14,15,16,17]. To integrate comprehensive metabolite profiling with compound isolation, the diethyl ether extract of the aerial parts was analyzed by LC–MS and, in parallel, subjected to column chromatography for the separation and purification of selected secondary metabolites. Subsequently, the total phenolic and flavonoid contents of this species, along with antioxidant activity, were evaluated in the diethyl ether and *n*-butanol extracts. Furthermore, falcarinol, identified as the major polyacetylene of this species, was evaluated for its cytotoxic effects on A375 human melanoma cells and HaCaT keratinocytes, suggesting differential sensitivity between cancerous and non-tumorigenic skin cells and highlighting its potential relevance for skin cancer research.

## 2. Results and Discussion

### 2.1. UHPLC–MS Phytochemical Profile

**Figure 1:**
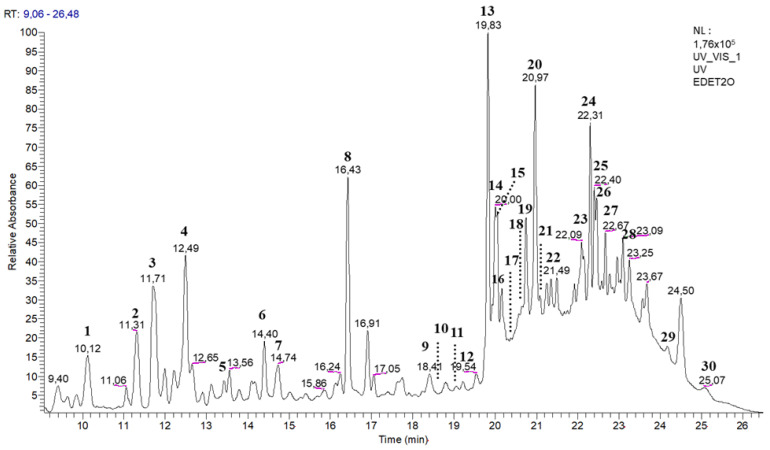
LC–UV chromatogram recorded at 280 nm of the *Eryngium dichotomum* diethyl ether extract.

UHPLC–MS analysis of the diethyl ether extract of the aerial parts of *Eryngium dichotomum* in negative ionization mode [M − H]^−^ revealed a rich and complex chromatographic profile (Figure 1), indicating the presence of numerous secondary metabolites (Table 1). Based on retention time, accurate mass measurements, predicted molecular formula, and comparison with previously published data, selected peaks were tentatively identified (see bold numbers in Figure 1). Interestingly, these peaks encompass diverse classes of natural products, such as phenolic acids, flavonoids and flavonoid glycosides, triterpenoid saponins, fatty acids, oxylipins, and polyacetylenes.

Phenolic acids were represented by peak **1**, identified as *p*-hydroxybenzoic acid (*m*/*z* 137, C_7_H_6_O_3_), and by peak **6**, corresponding to rosmarinic acid (*m*/*z* 359, C_18_H_16_O_8_). In addition, peak **13** was assigned to vanillic acid hexoside (*m*/*z* 329, C_14_H_18_O_9_). These compounds are widely reported in *Eryngium* species and are considered ubiquitous hydroxybenzoic and hydroxycinnamic acid derivatives within the genus. Their presence is consistent with earlier LC–MS studies on *E. alpinum*, and *E. planum*, and likely contributes to the pronounced antioxidant activity observed for the extract [15,16].

**Figure 2:**
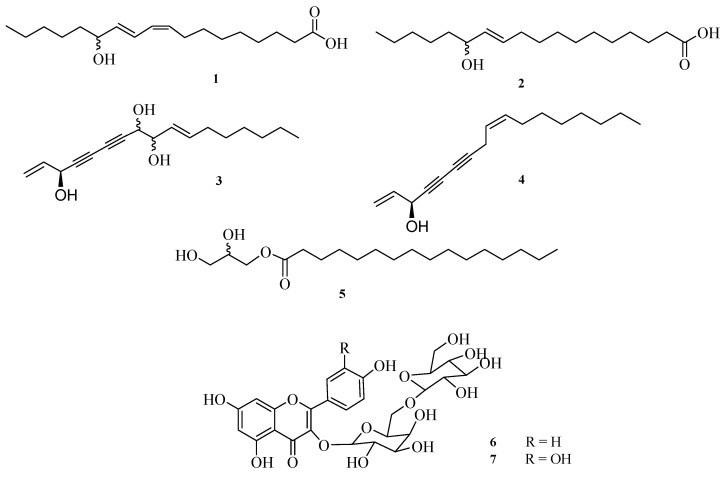
Identified compounds from the diethyl ether extract of *Eryngium dichotomum*.

Flavonoid compounds were represented by peaks **2** (*m*/*z* 625, C_27_H_30_O_17_) and **3** (*m*/*z* 609, C_27_H_30_O_16_), tentatively assigned to quercetin-*O*-dihexoside and kaempferol-*O*-dihexoside, respectively. These assignments were subsequently confirmed by 1D and 2D NMR spectroscopy, as kaempferol 3-*O*-β-D-glucopyranosyl-(1 → 6)-β-D-galactopyranoside (**6**) and quercetin 3-*O*-β-D-glucopyranosyl-(1 → 6)-β-D-galactopyranoside (**7**) (Figure 2). Additionally, other flavonoid glycosides could also be detected in peaks **5** (*m*/*z* 447, C_21_H_20_O_11_), **7** (*m*/*z* 431, C_21_H_20_O_10_) and **8** (*m*/*z* 593, C_27_H_30_O_15_), which were tentatively identified respectively quercetin-3-*O*-rhamnopyranoside (or kaempferol-3-*O*-glucopyranoside), kaempferol-7-*O*-rhamnopyranoside and kaempferol-7-*O*-rhamnopyranoside-3-*O*-glucopyranoside (or an acylated kaempferol glycoside bearing a *p*-coumaroyl moiety), respectively (Table 1). Although quercetin and kaempferol glycosides are typical chemotaxonomic markers of the *Eryngium* genus [18,19,20,21,22], this specific diglycoside substitution pattern is much less frequent than the rutinosides or monoglycosides commonly described in species such as *E. octophyllum* and *E. campestre* [22,23].

More complex acylated flavonoid glycosides were detected at later retention time, including peak **14** (*m*/*z* 769, C_40_H_34_O_16_), probably identified as a di-*p*-coumaroylated quercetin or isorhamnetin glycoside and peak **15** (*m*/*z* 739, C_39_H_32_O_15_), assigned to kaempferol-3-*O*-(2,6-di-*O*-*E*-*p*-coumaroyl)-β-D-glucopyranoside [24]. Such acylated flavonoids containing *p*-coumaroyl moieties have been previously described in *E. yuccifolium* and *E. campestre* [24,25]. It is nevertheless worth pointing that the molecular formula of peak **14** suggests the possible occurrence of a new flavonoid derivative, highlighting the chemical originality of *E. dichotomum*.

The LC–MS profile revealed the abundance of high-molecular-weight triterpenoid saponins, illustrated by a series of peaks starting with peak **9** (*m*/*z* 1057, C_52_H_82_O_22_), which was tentatively assigned to eryngioside E and its isomers eryngiosides G and N [24]. Further saponins were detected with peak **16** (*m*/*z* 1055, C_53_H_84_O_21_), identified as A1-barrigenol saponin [26], peak **17** (*m*/*z* 1041, C_52_H_82_O_21_) corresponding to eryngiosides F, H, or I, and peak **18** (*m*/*z* 1099, C_54_H_84_O_23_), assigned to eryngiosides J and M or saniculasaponins II and III [24]. Several additional peaks (**10**–**12**, **19**–**21**, and **22**) were attributed to unknown saponins, indicating the presence of structurally related but as yet uncharacterized triterpenoid glycosides. The detection of eryngiosides and saniculasaponins represents a key finding of this study. These oleanane-type triterpenoid saponins are regarded as characteristic constituents of *Eryngium* roots, particularly in species such as *E. yuccifolium* [24].

The apolar metabolites detected at higher retention times were attributed to fatty acids and notably to oxygenated C 17 polyacetylenes. Peaks **25**–**27** could be assigned to hydroxylated or trihydroxylated octadecatrienoic acid derivatives (HOA) with molecular formula ranging from C_18_H_30_O_3_ to C_18_H_30_O_5_ [14]. Peak **30** was assigned to two HOA-type oxylipins, namely (9*Z*,11*E*)-13-hydroxyoctadeca-9,11-dienoic acid (**1**) and (*E*)-13-hydroxyoctadec-11-enoic acid (**2**) (Figure 2). These attributions were confirmed by NMR upon isolation and comparison with literature data (see Section 2.2) [27,28]. Similarly, peaks **28** (*m*/*z* 275**,** C_17_H_24_O_3_) and **29** (*m*/*z* 243, C_17_H_24_O) were identified as respectively (*E*)-heptadeca-1,10-dien-4,6-diyne-3,8,9-triol (**3**) and falcarinol (**4**) (Figure 2), an assignment confirmed by NMR upon isolation [29,30,31]. Notably, falcarinol is a biologically active polyacetylene that has been widely reported in species of the *Eryngium* genus [7,10,30,31]. Finally, peak **31** (*m*/*z* 329, C_19_H_38_O_4_) was assigned to glycerol monopalmitate (**5**), a monoacylglycerol ester of palmitic acid, based on diagnostic NMR signals and mass spectrometry [32].

**Table 1:** Chemical composition of the diethyl ether extract of the aerial parts of *Eryngium dichotomum* by UHPLC–DAD–ESI/MS.

No	RT [min]	[M − H]^−^	Molecular Formula	UV Band (nm)	Tentatively Identified Compounds	Class ^a^	Ref.
**1**	10.12	137	C_7_H_6_O_3_	227,283	Hydroxybenzoic acid	PA	[33,34]
**2**	11.31	625	C_27_H_30_O_17_	256,367	Quercetin-3-*O*-dihexoside	F	[15], NMR
**3**	11.71	609	C_27_H_30_O_16_	266,321,339	Kaempferol-3-*O*-dihexoside	F.G	[2,15], NMR
**4**	12.49	387	-	321,237	Unknown	F/Ph	-
**5**	13.56	447	C_21_H_20_O_11_	252,367	Quercetin-3-*O*-rhamnopyranoside or kaempherol-3-*O*-glucopyranoside	F	[2]
**6**	14.4	359	C_18_H_16_O_8_	327	Rosmarinic acid	Ph	[15,33,35]
**7**	14.74	431	C_21_H_20_O_10_	233,341	Kaempferol-7-*O*-rhamnopyranoside	F	[2]
**8**	16.43	593	C_27_H_30_O_15_	236,266,314	kaempferol-7-*O*- rhamnopyranoside-3-*O*-glucopyranoside)/kaempferol-3*-O*-(2′-*O*-*Z*-*p*-coumaroyl)-D-glucopyranoside	FG	[2]
**9**	18.41	1057	C_52_H_82_O_22_		Eryngioside	S	[2]
**10**	19.08	1361		-	Unknown	S	-
**11**	19.22	1259		-	Unknown	S	-
**12**	19.54	1261		-	Unknown	S	-
**13**	19.83	329	C_14_H_18_O_9_	256,286	Vanilic acid hexoside	PhG	[15]
**14**	20.00	769	C_40_H_34_O_16_	234,268,312	Quercetin-3-*O*-(2-*O*-*E*-*p*-methoxycoumaroyl-6-*O*-*E*-*p*-coumaroyl)-D-gluco- pyranoside/Isorhamnetin 3-*O*-(2-*O*-*trans*-*p*-coumaroyl-6-*O*-*trans-p*-coumaroyl)-β-D-glucopyranoside	FG	-
**15**	20.04	739	C_39_H_32_O_15_	234,269,313	Kaempferol-3-*O*-(2,6-di-*O*-*E*-*p*-coumaroyl)-D-glucopyranoside	FG	[2,24]
**16**	20.58	1055	C_53_H_84_O_21_	-	3-*O*-D-glucopyranosyl(1 → 2)-[L-rhamnopyranosyl-(1→4)]-D-glucuronicpyranosyl-22-*O*-dimethylacryloyl-A1-barrigenol	S	[2]
**17**	20.59	1041	C_52_H_82_O_21_	-	Eryngioside	S	[2,36]
**18**	20.65	1099	C_54_H_84_O_23_	-	Eryngioside, Saniculasaponin	S	[2,36]
**19**	20.77	1097	-	-	Unkown	S	-
**20**	20.97	585 1045	-	238,297,320 -	Unkown Unkown	F S	-
**21**	21.25	883	-	-	Unkown	S	-
**22**	21.49	577	-	-	Unkown	S	-
**23**	22.09	555	C_35_H_56_O_5_	-	Derivative of barregenol -A_2_ with angeloyl group	TT	[2]
**24**	22.31	557	C_35_H_58_O_5_ C_35_H_56_O_5_	-	Derivative of barregenol -A_2_ with acetyl group	TT	[2]
		555		-			
**25**	22.4	325	C_18_H_30_O_5_	271	Unknown trihydroxy fatty acid isomer	FA	-
**26**	22.46	325	C_18_H_30_O_5_	273	Unknown trihydroxy fatty acid isomer	FA	**-**
**27**	22.67	293	C_18_H_30_O_3_	247,275	Unknown hydroxyoctadeca-trienoic acid	FA	**-**
**28**	22.98	275	C_17_H_24_O_3_	244	(*E*)-heptadeca-1,10-dien-4,6-diyne-3,8,9-triol	PoAc	[29], NMR
**29**	24.17	243	C_17_H_24_O	204,252	(*Z*),1,9-heptadecadiene-4,6-diyn-3-ol (Falcarinol)	PoAc	[30], NMR
**30**	24.5	295	C_18_H_32_O_3_	N.D	(*Z*, *E*)-13-hydroxyoctadeca-9,11-dienoic acid	FA	[27,28], NMR
		297	C_18_H_34_O_3_	N.D	(*E*)-13-hydroxyoctadec-11-enoic acid		
**31**	25.07	329	C_19_H_38_O_4_	N.D	Glycerol monopalmitate	MG	[32], NMR

^a^ Class of compounds: PhA—phenolic acid, PhG—phenolic glycoside, F—flavonoids, FG—flavonoids glycosides, Ph—phenol, S—Saponin, TT—triterpenoid, FA—Fatty acid, PoAc—polyacetylene, MG—monoglyceride, and N.D—not determined.

### 2.2. Isolation and Structure Elucidation of Compounds ***1***–***7***

From the above-mentioned LC–MS analysis of the diethyl ether extract of *Eryngium dichotomum*, several interesting and new compounds were possibly identified. We therefore attempted to isolate some of them. Compounds **1**–**7** (Figure 2) could be obtained in pure form and fully characterized (see Section 3.3). The isolated compounds were subsequently correlated with LC–MS peaks **2**, **3**, **28**, **29**, and **30**, and their structures were elucidated using ^1^H, ^13^C, and 2D NMR experiments (HSQC, HMBC, and COSY) (see Supplementary Materials), in combination with mass spectrometric data and comparison with literature reports. These results confirm the reliability of the tentative LC–MS-based identifications.

For the oxylipins associated with peak **30**, compound **2**, deduced as (*E*)-13-hydroxyoctadec-11-enoic acid, was successfully isolated in pure form as colorless oil, whereas compound **1**, namely (*Z*,*E*)-13-hydroxyoctadeca-9,11-dienoic acid, was isolated as a mixture with compound **2** in an approximately 1:1 ratio, as supported by ^1^H NMR and mass spectra (see Supplementary Materials). Indeed, the analysis of both spectra for compounds **1** and **2** confirmed that they are C_18_ oxylipins featuring a hydroxylated methine at C-13, along with a terminal methyl group resonating at δ_H_ 0.88 (t, *J* = 6.9 Hz, H-18). In ^1^H NMR spectrum of compound **1**, four olefinic proton signals were observed at δ_H_ 6.48 (dd, *J* = 15.2, 11.1 Hz, H-11), 5.97 (t, *J* = 11.2 Hz, H-10), 5.66 (dd, *J* = 15.2, 6.9 Hz, H-12), 5.45 (dt, *J* = 10.8, 7.8 Hz, H-9), indicating the presence of a conjugated diene system (Δ^9,11^) with mixed (*Z*,*E*) geometry, as supported by the coupling constants (*J* = 15.2 and 11.2 Hz). In contrast, compound **2** displayed only two olefinic protons at δ_H_ 5.62 (dd, *J* = 15.2, 6.9 Hz, H-11), 5.44 (dd, *J* = 15.2, 6.9 Hz, H-12), consistent with a trans double bond (Δ^11^). The oxymethine proton at C-13 appeared at δ_H_ 4.16 (q, *J* = 6.4 Hz) in compound **1** and slightly upfield at δ_H_ 4.03 (d, *J* = 6.6 Hz) in compound **2**, reflecting differences in the neighboring unsaturation pattern. The remaining aliphatic protons in both compounds appeared as multiplets in the δ_H_ 1.74–1.17 region, while signals around δ_H_ 2.3 were assigned to methylene groups adjacent to the carboxylic function (H-2). Compound **2** exhibited a deprotonated molecular ion at *m*/*z* 297.25 [M − H]^−^ in its ESI–MS spectrum, consistent with the molecular formula C_18_H_34_O_3_ and representing a 2 Da increase compared to compound **1** (*m*/*z* 295.23 [M − H]^−^). The characteristic ESI–MS base peak at *m*/*z* 197.81 observed for both compounds is diagnostic of 13-hydroxyoctadecanoid (13-HODE-type) derivatives and clearly differentiates them from the corresponding 9-hydroxy isomers [27,28,37]. Notably, compound **1,** commonly referred to as coriolic acid, is a relatively common oxidation product of linoleic acid in the *Eryngium* genus [2]. In contrast, its monounsaturated analogue **2** has been reported much less frequently as a natural constituent in plant species.

Compound **3**, corresponding to peak **28**, and representing the first report of this metabolite within the *Eryngium* genus, was identified as (*E*)-heptadeca-1,10-dien-4,6-diyne-3,8,9-triol. The ^1^H NMR spectrum of this compound was consistent with a C-17 polyacetylene skeleton, showing a terminal methyl group at δ_H_ 0.92 (t, *J* = 6.9 Hz, H-17), along with signals characteristic of a terminal olefin at δ_H_ 5.40 (dt, *J* = 17.1, 1.3 Hz, H-1a), 5.19 (dt, *J* = 10.2, 1.3 Hz, H-1b), and 5.93 (ddd, *J* = 17.1, 10.2, 5.3 Hz, H-2) (see Supplementary Materials). The spectrum also exhibited a trihydroxylated pattern, evidenced by three oxymethine protons at δ_H_ 4.89 (m, H-3), 4.19 (br d, *J* = 6.8 Hz, H-8), and 3.97 (br t, *J* = 6.8 Hz, H-9). An internal double bond was assigned at (Δ^10^) based on the olefinic resonances at δ_H_ 5.52 (dd, *J* = 15.4, 6.8 Hz, H-10) and 5.80 (dt, *J* = 15.4, 6.8 Hz, H-11). The large coupling constant (*J* = 15.4 Hz) is indicative of a trans configuration. The proposed structure was further supported by ESI–MS data, which showed a pseudomolecular ion peak at *m*/*z* 299.16 [M + Na]^+^, consistent with the molecular formula C_17_H_24_O_3_. This polyacetylene was previously reported for an Apiaceae species, *Glehnia littoralis* ssp. *Leiocarpa* [29]. In contrast, compound **4** was identified as the major constituent of the diethyl ether extract. Also known as falcarinol ((*Z*)-heptadeca-1,9-dien-4,6-diyn-3-ol), this compound is a well-documented polyacetylene, characteristic of *Eryngium* species.

Compound **5** is an unexpected glycerol-derived compound. Its structural elucidation was achieved by detailed NMR spectroscopic and mass spectrometry analysis, which confirmed its glycerol monopalmitate structure (see Supplementary Materials). Although newly found in *Eryngium* species, the occurrence of glycerol-derived compounds is consistent with the chemical features of the Apiaceae family. Many Apiaceae species are indeed characterized by glycerol-based lipids, such as triacylglycerols containing palmitic and other fatty acids [38,39].

In addition, two flavonoid glycosides bearing galactose and glucose moieties were isolated and structurally characterized by NMR and mass spectrometric analyses as kaempferol 3-*O*-β-D-glucopyranosyl-(1 → 6)-β-D-galactopyranoside (**6**) and quercetin 3-*O*-β-D-glucopyranosyl-(1 → 6)-β-D-galactopyranoside (**7**) (see Supplementary Materials). These flavonoids have been previously reported only once within the genus *Eryngium* from *Eryngium tricuspidatum* [40].

### 2.3. Total Phenolic and Flavonoids Content

Previous studies on *Eryngium* species have mainly showed the richness of this genus in polyphenols, in particular phenolic acids and flavonoids. However, direct comparison of the data reported for total phenolic and flavonoid contents (TPC, TFC) remains challenging due to variations within a species itself [40], but also in extraction conditions, and sometimes in inconsistencies in the units used to report phenolic and flavonoid contents [41,42,43,44,45]. As this study represents to the best of our knowledge the first evaluation of the total phenolic and flavonoid contents (TPC, TFC) of *Eryngium dichotomum*, variability of the species cannot be evaluated.

In the present study, the dried aerial parts of *E. dichotomum* were extracted with a 8:2 mixture of acetone and water. Upon evaporation, the resulting aqueous solution was taken up with diethyl ether and then *n*-butanol (See Section 3.3). Both extracts were then used to evaluate the total phenolic and flavonoids contents (Table 2) using classical methods (See Section 3.4 and Section 3.5). The results are expressed in mg of quercetin equivalent per gram of dry extract (mg QE/g).

Our results showed that the *n*-butanol extract exhibited the highest TPC (366.42 mg GAE/g) and TFC (231.17 mg QE/g), significantly higher compared to levels of the diethyl ether extract (TPC: 194.89 mg GAE/g; TFC: 96.80 mg QE/g). This difference can be attributed to the polarity of the solvents, which influences the distribution of total polyphenolic compounds, resulting in lower concentrations in diethyl ether extract (low polarity) and higher concentrations in *n*-butanol extract (high polarity).

These results revealed that both the TPC and TFC in *E. dichotomum* were higher than the results reported in litterature for other *Eryngium* species. Bouzidi et al. [42] reported a TPC of 27.77 µg GAE/mg for the ethyl acetate extract of the *Eryngium campestre* aerial parts, and a TFC of 7.54 µg QE/mg for the aqueous root extract. Similarly, Nabavi et al. [43] reported for the leaves of *Eryngium caucasicum* a TPC and TFC of 62.3 mg GAE/g extract, and 25.3 mg QE/g extract, respectively. In another study, the ethyl acetate extract of *Eryngium kotschyi* exhibited the TPC of 173.71 mg GAE/g extract and the TFC of 86.98 mg catechin equivalents (CE)/g extract) [44]. More recently, a study on *Eryngium billardieri* indicated a TPC of 19.25 mg GAE/g extract in the hydromethanolic extract of the flowers [45]. TPC of *E. dichotomum* polar extract is thus around twice more important than in *E. kotschyi*, which exhibits the second highest content, and up to ninteen times higher than in *E. billardieri*, which exhibits the lowest content.

The TPC of *E. dichotomum* polar extract is approximately twofold higher than that of *E. kotschyi*, which exhibits the second highest content, and up to nineteen fold higher than that of *E. billardieri*, which shows the lowest value. This variation may be attributed to several factors. First, the polarity of *n*-butanol favors the extraction of moderately polar phenolic compounds, leading to their enrichment in this fraction compared to less polar or more aqueous extracts. Additionally, differences in phenolic content among *Eryngium* species may be influenced by environmental and ecological conditions, including geographic origin, climate, and soil composition.

### 2.4. In Vitro Biological Assays

#### 2.4.1. Antioxidant Activity

Several studies conducted on different *Eryngium* species have shown that these plants could represent promising sources of antioxidant agents. However, these reported antioxidant activities varied according to the assay systems used [41,44,45,46].

In the present study, the antioxidant activity of *E. dichotomum* extracts was evaluated using classical methods, i.e., DPPH, ABTS scavenging methods, and FRAP (see Section 3.6).

The results presented in Table 3 demonstrated that *E. dichotomum* extracts have significant antioxidant activity. The diethyl ether extract exhibits a slightly lower DPPH activity than the *n*-butanol extract, as indicated by the higher IC_50_ values respectively 21.23 and 18.36 μgTrolox/g. In contrast, the diethyl ether extract extract exhibited significantly higher reducing power FRAP (164.11 mg AA/g), suggesting a greater ability to reduce ferric ions. In the ABTS assay, the *n*-BuOH extract showed more pronounced activity, with a lower IC_50_ value (94.15 μgTrolox/g). Compared to the standard antioxidants, namely Trolox and ascorbic acid, both extracts exhibit lower activity, but nevertheless demonstrate interesting antioxidant potential.

Our results are consistent with previous findings. For an Algerian *E. campestre*, the *n*-butanol extract of the aerial parts exhibited strong antioxidant activity in the DPPH assay with a low IC_50_ value (16.14 μg/mL) [42]. In another study carried out on the *E. caucasicum* at flowering stage, methanolic extracts exhibited different levels of antioxidant activity across the tested models, but in the DPPH assay, the leaf extract demonstrated a strong radical-scavenging activity (0.15 mg/mL) [47].

The antioxidant activity observed in *E. dichotomum* extracts may be attributed to the diversity of flavonoids and phenolic compounds identified by UHPLC–DAD–ESI/MS. Although these constituents were primarily detected in the diethyl ether extract, structurally related flavonoids and phenolic derivatives are also likely present in the more polar *n*-butanol extract, but with different relative abundances and higher polarity.

Although the *n*-BuOH extract exhibited superior radical scavenging activity in both DPPH and ABTS assays, the Et_2_O extract showed markedly higher reducing power in the FRAP assay. The *n*-BuOH fraction is likely enriched in more polar phenolic constituents that are particularly effective in DPPH and ABTS radical scavenging assays, whereas the Et_2_O extract may contain a higher proportion of less polar metabolites, including oxylipins and fatty acid-derived compounds, which exhibit stronger electron-donating properties and thus preferentially enhance ferric ion reducing activity.

Phenolic acids such as vanillic acid, *p*-hydroxybenzoic acid, and rosmarinic acid, which are well documented for their free radical scavenging abilities, are known to contribute significantly to the DPPH and ABTS assay responses [48,49,50]. In addition, flavonoids, particularly derivatives of quercetin, kaempferol, and isorhamnetin, possess strong antioxidant activity via electron donation and metal chelation mechanisms [51,52].

Triterpene saponins, such as eryngiosides and baregenol derivatives identified in the diethyl ether extract, have also been associated with notable antioxidant activity in complex plant extracts [53,54]. Furthermore, the presence of hydroxylated fatty acids and polyacetylenes suggests synergistic effects that could enhance the overall activity of the extracts [55]. Thus, the combination of these classes of compounds explains the measured antioxidant activity and justifies the use of multiple methods for a comprehensive evaluation.

#### 2.4.2. Cytotoxicity of Falcarinol in A375 Melanoma Cells and HaCaT Keratinocytes

Polyacetylene natural products are known to exhibit interesting bioactivities including anti-inflammatory, antiplatelet-aggregatory, cytotoxic, and antitumor activity [56]. Among them, falcarinol has been shown to inhibit the formation of polyps and tumors in rat colon [57]. Such anti-proliferative effect has also been observed against human pancreatic carcinoma cells, but not against normal pancreatic cells [3,58], as well as on leukeamia cell lines [59]. It is thus worth exploring other anti-cancer activity due to famcarinol.

**Figure 3:**
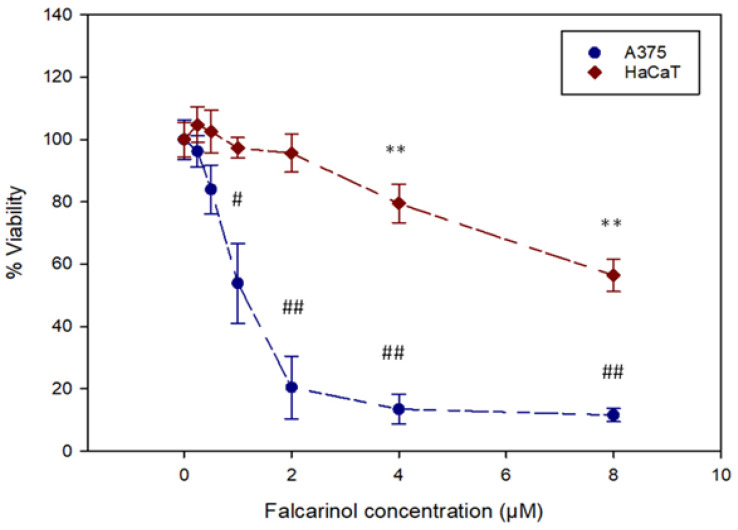
Effect of Falcarinol on the viability of A375 amelanotic melanoma cells and HaCaT keratinocytes after 24 h. Results are expressed as the mean ± standard deviation (SD). Statistical analysis was performed using one-way ANOVA followed by Dunn’s multiple comparison test for A375 cells and Dunnett’s multiple comparison test for HaCaT cells. Statistical significance relative to the control is indicated as follows: ** *p* ≤ 0.001 for the HaCaT cell line; # *p* < 0.05, ## *p* < 0.001 for the A375 cell line.

**Figure 4:**
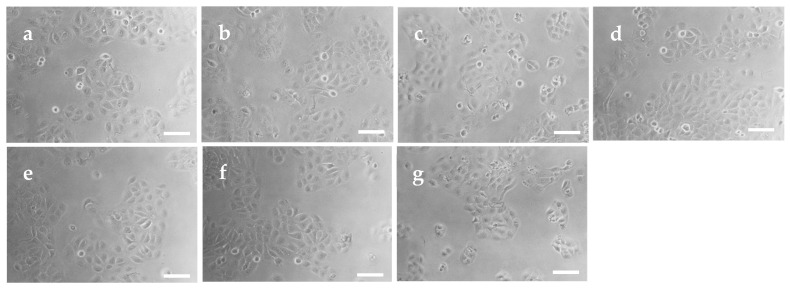
Light microscopy images (×100) of HaCaT cells exposed to Falcarinol for 24 h: (**a**) 0; (**b**) 0.25; (**c**) 0.5; (**d**) 1; (**e**) 2; (**f**) 4; and (**g**) 8 µM of Falcarinol. Bar corresponds to 100 μm.

**Figure 5:**
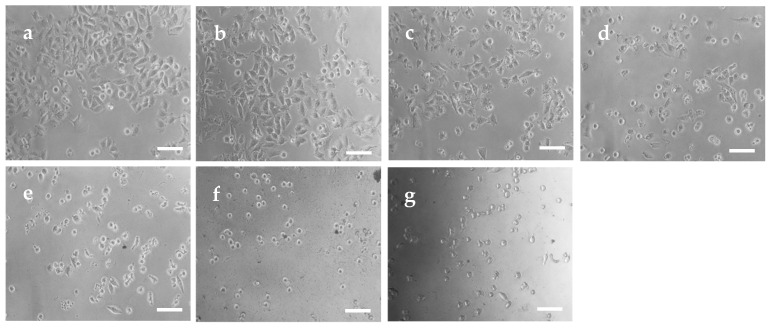
Light microscopy images (×100) of A375 cells exposed to Falcarinol for 24 h: (**a**) 0; (**b**) 0.25; (**c**) 0.5; (**d**) 1; (**e**) 2; (**f**) 4; and (**g**) 8 µM of Falcarinol. Bar corresponds to 100 μm.

In this context, the cytotoxic effects of falcarinol on A375 melanoma cells and HaCaT keratinocytes (non-tumorigenic) were evaluated by the MTT assay after 24 h of exposure (Figure 3, Figure 4 and Figure 5). A375 cells showed a large decrease in viability to 53.9% at 1 µM concentration (*p* < 0.05), while HaCaT keratinocytes demonstrated a marked resistance to the same concentration of Falcarinol. Although a significant reduction to 79.5% was observed at 4 µM (*p* < 0.001), HaCaT keratinocytes only reached the same relative level of viability as A375 melanoma cells at 8 µM (56.4% viable) (*p* < 0.001), approximately 8-fold higher than A375 melanoma cells at 1 µM. These findings indicate that A375 melanoma cells are more sensitive to falcarinol exposure than non-tumorigenic HaCaT keratinocytes, particularly at lower concentrations.

The IC_50_ values (Table 4) further support the differential cytotoxicity of falcarinol between A375 melanoma cells and HaCaT keratinocytes. Specifically, the IC_50_ of A375 melanoma cells was much lower (1.10 µM) compared to that of HaCaT keratinocytes (9.03 µM), approximately 8-fold difference, demonstrating higher sensitivity of melanoma cells Falcarinol, indicating potential therapeutic relevance.

This preferential cytotoxic activity of falcarinol is further supported by the existing literature. For instance, Cheung et al. showed that falcarinol-type polyacetylenes target human pancreatic carcinoma while producing no significant effect on the normal human pancreatic cell lines [58]. Furthermore, the present study extends and reinforces the broad-spectrum antitumoral activity of falcarinol, already substantiated by multiple studies in different types of cancers, including human glioblastoma, leukemia, lymphoma, and myeloma [60,61]. Mechanistically, the broad-spectrum antitumoral effects of falcarinol and related polyacetylenes are mediated by interactions with multiple molecular targets and signaling pathways. Previous studies have shown that these compounds can act as alkylating agents, inducing DNA damage, inhibiting mitochondrial respiration, and modulating the activity of key cellular enzymes, such as Na+/K+-ATPase [62]. Furthermore, falcarinol induces apoptosis and cell cycle arrest by modulating the expression of apoptosis-related proteins (such as downregulating XIAP and Cytochrome C), inducing endoplasmic reticulum stress, and interacting with nuclear receptors, including the peroxisome proliferator-activated receptor gamma (PPARγ) [63,64].

## 3. Materials and Methods

### 3.1. General Experimental Procedures

#### 3.1.1. UHPLC–MS

Extract samples were suspended in 1 mL of methanol and 5 μL was injected into an Ultimate 3000 system (Dionex Co., San Jose, CA, USA) which was integrated with a UV detector (Dionex Co., San Jose, CA, USA) and a Thermo LTQ XL mass spectrometer (Thermo Scientific, San Jose, CA, USA), featuring an electrospray ionization (ESI) interface for analysis. A Hypersil Gold C18 column (100 mm length, 2.1 mm internal diameter, 1.9 μm particle size, end-capped; Thermo Scientific, San Jose, CA, USA) was employed for separation at 25 °C. The used mobile phase was a mixture of acetonitrile (A) and formic acid in water (B) injected at a flow rate of 0.2 mL/min. The gradient of B was maintained at 5% for the initial 14 min, increased to 40% over 2 min, reaching 100% over the subsequent 7 min. At 23 min, it was reset to 5% until the end. An electrospray ionization (ESI) source at 5.00 kV was used in negative-ion mode, with a capillary temperature of 275 °C. A mass range of 100 to 2000 *m*/*z* was encompassed. Spectral data collection covered the 200–500 nm UV–vis range, with chromatographic profile captured at 280 nm. Spectra processing utilized the Thermo Xcalibur Qual Browser data system (Thermo Scientific, San Jose, CA, USA), and the identification of peaks was achieved through a comparative analysis of retention times, UV–vis spectra and MS spectral data from previously described literature.

#### 3.1.2. Spectroscopy

NMR spectra were obtained on a Bruker 500 (Bruker Avance NEO spectrometer) (Bruker Optic, Ettingen, Germany), with measurements performed at 500.130 MHz for ^1^H and 125.758 MHz for ^13^C. The spectrometer was equipped with a dual ^1^H/^13^C helium cryoprobe (5 mm CP DCH ^13^C/^1^H/D z-grad). Alternatively, spectra were recorded on a 500 MHz Bruker Avance III HD spectrometer (Bruker Biodpin, Ettingen, Germany) (500.070 MHz for ^1^H and 125.743 MHz for ^13^C), equipped with a helium cryoprobe BBO (5 mm CP BBO 500S1 BBF-H-D-05z), and additionally on a 300 MHz Avance III spectrometer (Bruker Optic, Ettingen, Germany), with measurements performed at 300.130 MHz for ^1^H. This spectrometer was equipped with a BBO probe (5 mm PABBO BB-1H/D z-grad). The spectra were recorded in deuterated chloroform and methanol. Chemical shifts (δ) are in part per million (ppm) relative to tetramethylsilane (TMS). Referenced solvent peaks of CDCl_3_ and CD_3_OD (Eurisotop, a Cambridge Isotope Laboratories company, Saint-Aubin, France): ^1^H δ 7.26 ^13^C 77.22 ^1^H δ 3.31, ^13^C δ 49.1 respectively.

Mass spectrometry (MS) experiments were performed on an Orbitrap Exploris MX (Thermo Fisher Scientific, Bremen, Germany) equipped with heated electrospray ionization (HESI-II) sprayer. Calibration was performed using Pierce FlexMix Calibration Solution in positive mode and negative mode (Thermo Fisher Scientific, San Jose, CA, USA). Sample solutions were introduced into the spectrometer source with Vanquish autosampler (Thermo Fisher, Bremen, Germany). Complementary analyses were carried out on a Bruker Daltonics microTOF spectrometer (Bruker Dal-tonik GmgH, Bremen, Germany) equipped with an orthogonal electrospray (ESI) interface. Calibration was performed using Tuning mix (Agilent Technologies, Santa Clara, CA, USA). Sample solutions were introduced into the spectrometer source with a syringe pump (Harvard type 551111: Harvard Apparatus Inc., South Natick, MA, USA) at a flow rate of 4 µL.min^−1^.

#### 3.1.3. Chromatography

Analytical TLC was performed on precoated SiO_2_ plates (Merck Kieselgel 60 F254, 0.25 and 0.5 mm (Merck KGaA, Darmstadt, Germany), with detection provided by UV light (254 nm) and by spraying with a mixture of sulfuric and acetic acids reagents followed by heating (120 °C).

SiO_2_ column chromatography was performed using Merck Kieselgel 60 (70–230 mesh). Sephadex LH-20 was from Amersham Pharmacia Biotech (Uppsala, Sweden), and RP-18 silica cartridges of the Chromabond^®^ C18 type and Chromabond^®^ C18 Hydra were from Macherey-Nagel, Düren, Germany.

### 3.2. Plant Material

Aerial parts of *Eryngium dichotomum* Desf. (Apiaceae) were collected in Batna, eastern Algeria, during the flowering period in May 2019. The plant was identified by Prof. Oudjehih Bachir from the Department of Agronomy, Institute of Veterinary and Agronomic Sciences, University of Batna 1. A voucher specimen (No. 700/LCCE) was deposited in the Herbarium of the University of Batna 1, Batna, Algeria. The collected material was air-dried at room temperature and subsequently ground using a laboratory mill.

### 3.3. Extraction and Isolation

One kilogram of the dried aerial parts of *Eryngium dichotomum* was extracted at room temperature (25 °C) using 10 L of 80% acetone in water. The extract was concentrated to remove the solvent and the aqueous phase (600 mL) was subjected to successive liquid–liquid extraction with diethyl ether (Et_2_O, 2 L) and *n*-butanol (BuOH, 1 L). After removal of the solvents under reduced pressure, two organic fractions were obtained: 21 g from Et_2_O and 27 g from *n*-BuOH. A portion (15.0 g) of the diethyl ether extract was subjected to fractionation by column chromatography on silica gel. Elution was performed using a gradient of increasing polarity, starting first with hexane/Et_2_O (95/5 *v*/*v*) mixture, the polarity was gradually increased by adding 10% diethyl ether in hexane in 100 mL increments until reaching 100% diethyl ether. Further separation was achieved via gradient elution with CHCl_3_/MeOH ranging from (90/10, *v*/*v*) to pure methanol. Fractions were monitored by thin layer chromatography (TLC) and combined according to their chromatographic similarity, affording a total of 23 final fractions designated as Fr1-23. Fraction Fr14 (167.5 mg) was selected on the basis of its high fatty acid content, as indicated by its TLC chromatographic profile. Fractionation of Fr14 by silica gel column chromatography, using a gradient of increasing polarity (hexane/EtOAc), yielded 13 subfractions (Fr14-1 to Fr14-13). Subfraction Fr14-7 (30 mg) was further purified on a reversed-phase solid-phase (RP-18 SPE) cartridge using a MeOH/H_2_O gradient (0–100% MeOH). Eluted fraction with MeOH/H_2_O 8:2) afforded pure compound **1** (2 mg, Rf = 0.33) whereas fraction eluted with MeOH/H_2_O 9:1 yielded compound **2** (1 mg, Rf = 0.36). Purification of subfraction Fr14–10 (60 mg) was performed on Sephadex LH-20, using as eluent system a mixture of CHCl_3_/MeOH (1/1, *v*/*v*), obtaining 8 subfractions (Fr14-10-1 to Ed14-10-8). Combined subfractions Fr14-10-2, Fr14-10-3, and Fr14-10-4 (13 mg) was subjected to purification using RP-18 SPE cartridge, with MeOH/H_2_O (70/30, *v*/*v*) as eluent, affording compound **3** (1.4 mg, Rf = 0.22). Fraction **Fr11** (60 mg) obtained from the diethyl ether extract fractionation was chromatographed on Sephadex LH-20 column using isocratic elution CHCl_3_/MeOH (1/1, *v*/*v*), directly providing compound **4** (20 mg, Rf = 0.28). Subsequently, fraction Fr18 (450 mg) was further purified by silica gel column chromatography using two successive solvent systems: first, a hexane/EtOAc gradient (95:5 to 100% EtOAc), followed by an EtOAc/MeOH gradient (90:10 to 100% MeOH), to give 15 subfractions (Fr18-1 to Fr18-15). Combined subfractions Fr18-6, Fr18-7, and Fr18-8 (172 mg) was rechromatographed on a silica gel column using a Hexane/EtOAc gradient as eluent, resulting in 26 further subfractions (A1–A26). Selected fraction A23 (9.7 mg) was purified using RP-18 SPE cartridge, eluted with MeOH/H_2_O (90/10, *v*/*v*) to give pure compound **5** (4.5 mg, Rf = 0.46). The more polar fraction of the diethyl ether extract (Fr23, 2 g) was subjected to further purification based on TLC analysis showing a high flavonoid content. Silica gel column chromatography was performed using CHCl_3_/MeOH as eluent, yielding 12 subfractions (Fr23-1 to Fr23-12). Subfraction Fr23-10 (326 mg) was subjected to column chromatography on Sephadex LH20 using isocratic system solvent (CHCl_3_/MeOH, 1/1, *v*/*v*). This fractionation process resulted in the isolation of 11 subfractions (Fr23-10-1 to Fr23-10-11). Subfraction Fr23-10-6 (175.4 mg) was further purified by (RP-18 SPE) using elution system gradient of methanol in water (0, 10, 30, 50, 100%), affording pure compound **6** (4 mg, Rf = 0.5). Similarly, subfraction Fr23-10-8 (33 mg) yielded pure compound **7** (2 mg, Rf = 0.36).

The spectroscopic characteristics of all isolated compounds are summarized below.

Compounds **1** and **2** (Peak **30**):

(*Z*, *E*)-13-hydroxyoctadeca-9,11-dienoic acid (**1**), colorless oil.
^1^H NMR (500 MHz, CDCl_3_) δ_H_ 6.48 (dd, *J* = 15.2, 11.1 Hz, 1H, H-11), 5.97 (t, *J* = 11.2 Hz, 1H, H-10), 5.66 (dd, *J* = 15.2, 6.9 Hz, 1H, H-12), 5.45 (dt, *J* = 10.8, 7.8 Hz, 1H, H-9), 4.16 (q, *J* = 6.43 Hz, 1H, H-13), 2.32 (br s, 2H, H-2), 2.17 (q, *J* = 7.28 Hz, 2H, H-8), 1.74–1.17 (m, 18H), 0.88 (t, *J* = 6.9 Hz, 3H, H-18). ESI–MS: positive mode *m*/*z* 319.22 [M + Na]^+^, negative mode *m*/*z* 295.23 [M − H]^−^, 195.81 (base peak).
(*E*)-13-hydroxyoctadec-11-enoic acid (**2**), colorless oil.
^1^H NMR (500 MHz, CDCl_3_) δ_H_ 5.62 (dd, *J* = 15.2, 6.9 Hz, 1H, H-11), 5.44(dd, *J* = 15.2, 6.9 Hz, 1H, H-12), 4.03 (d, *J* = 6.6 Hz, 1H, H-13), 2.34 (t, *J* = 7.5 Hz, 2H, H-2), 2.02 (d, *J* = 6.8 Hz, 2H, H-10), 1.74–1.17 (m, 18H), 0.88 (t, *J* = 6.9 Hz, 3H, H-18). ESI–MS: positive mode *m*/*z* 321.24 [M + Na]^+^, negative mode *m*/*z* 297.24 [M − H]^−^, 197.81 (base peak).
Compound **3** (peak **28**): (*E*)-heptadeca-1,10-dien-4,6-diyne-3,8,9-triol, colorless oil.
^1^H NMR (500 MHz, MeOD) δ_H_ 5.40 (dt, *J* = 17.1, 1.3 Hz, H-1a), 5.19 (dt, *J* = 10.2, 1.3 Hz, H-1b), 5.93 (ddd, *J* = 17.1, 10.2, 5.3 Hz, H-2), 4.89 (m, H-3, overlapped with residual solvent signal), 4.19 (br d, *J* = 6.8 Hz, H-8), 3.97 (br t, *J* = 6.8 Hz, H-9), 5.52 (dd, *J* = 15.4, 6.8 Hz, H-10), 5.80 (dt, *J* = 15.4, 6.8 Hz, H-11), 2.08 (m, H-12), 1.42 (m, H-15), 1.32 (m, H-16), 1.31 (m, H-13, H-14), 0.92 (t, *J* = 6.9 Hz, H-17). ^13^C NMR (100 MHz, CDCl_3_) δ_C_, 136.6 (C-2), 134.5 (C-11), 127.7 (C-10), 115.3 (C-1), 78.5 (C-7), 77.8 (C-4), 75.2 (C-9), 68.9 (C-6), 68.7 (C-5), 66.2 (C-8), 62.4 (C-3), 32.1 (C-12), 31.5 (C-13), 29.4 (C-14), 28.8 (C-15), 22.3 (C-16), 13.4 (C-17). ESI–MS: positive mode *m*/*z* 299.16 [M + Na]^+^.
Compound **4** (peak **29**): (Z)-heptadeca-1,9-dien-4,6-diyn-3-ol (Falcarinol), colorless oil.
^1^H NMR (CDCl_3_, 500 MHz) δ_H_ 5.93, (1H, ddd, *J* = 15.9, 10.3, 5.4 Hz, H-2), 5.40 (m, H-9), 5.50 (m, H-10), 3.01 (2H, d, *J* = 6.8 Hz, H-8), 5.47 (1H, d, *J* = 16.5 Hz, H-1a), 5.25 (1H, dd, *J* = 16.5, 10.3 Hz, H-1b), 4.92 (1H, d, *J* = 5.4 Hz, H-3), 2.05–1.27 (multiplet m, H-11 to H-16), 0.88 (3H, t, *J* = 7.1 Hz, H-17). ESI–MS: negative mode *m*/*z* 243.85 [M − H]^−^.
Compound **5** (peak **31**): Glycerol monopalmitate, white powder.
^1^H NMR (500 MHz, CDCl_3_) δ_H_ 4.18, (dd, *J* = 5.9, 11.9 Hz, 1H, CH_2_OCO, 3.93 (t, *J* = 5.06 Hz, 1H, CHOH), 3.70 (dd, *J* = 4.5, 11.5 Hz, 1H, CHOH), 3.60 (dd, *J* = 5.6, 11.5 Hz, 1H, CHOH), 2.35 (t, *J* = 7.6 Hz, 2H, CH_2_CO), 1.63 (t, *J* = 7.06 Hz, 2H, CH_2_), 1.26 (m, 24H, 12CH_2_), 0.88 (t, *J* = 7.0 Hz, 3H, CH_3_). ESI–MS: negative mode *m*/*z* 329.27 [M − H]^−^, positive mode *m*/*z* 353.27 [M + Na]^+^.
Compound **6** (peak **3**): Kaempferol 3-*O*-β-D-glucopyranosyl-(1 → 6)-*O*-β-D-galactopyranoside, amorphous yellow powder.
^1^H NMR (500 MHz, CD_3_OD) δ_H_ 8.13 (d, *J* = 8.9 Hz, H-2′/H-6′), 6.89 (d, *J* = 9.0 Hz, H-3′/H-5′), 6.43 (d, *J* = 2.0 Hz, H-8), 6.22 (d, *J* = 2.0 Hz, H-6), 5.12 (d, *J* = 7.7 Hz, H-1″), 4.12 (d, *J* = 7.8 Hz, H-1‴), 3.89 (t, *J* = 4.1 Hz, H-4″), 3.87 (t, *J* = 4.2 Hz, H-6″a), 3.84 (dd, *J* = 8.9, 4.7 Hz, H-2″), 3.80 (dd, *J* = 9.8, 2.4 Hz, H-6‴a), 3.67 (m, H-5″), 3.66 (m, H-6″b), 3.63 (dd, *J* = 7.5, 5.8 Hz, H-6‴b), 3.56 (dd, *J* = 9.5, 3.5 Hz, H-3″), 3.21 (t, *J* = 9, H-5‴), 3.17 (t, *J* = 9, H-3‴), 3.10 (m, H-4‴), 3.08 (t, *J* = 8.8Hz, H-2‴). ^13^C NMR (125 MHz, CD_3_OD) δ_C_ 164.6 (C-7), 161.7 (C-5), 160.6 (C-4′), 158.3 (C-2), 157.3 (C-9), 131.1 (C-2′), 122.5 (C-6′), 121.4 (C-1′), 115.1 (C-3′), 115.1 (C-5′), 103.6 (C-1″), 103.3 (C-1‴), 98.3 (C-6), 93.2 (C-8), 76.4 (C-3‴), 75.6 (C-4‴), 74.3 (C-5″), 73.5 (C-3″), 73.4 (C-2‴), 71.9 (C-2″), 70.1 (C-5‴), 68.7 (C-4″), 67.7 (C-6″), 61.3 (C-6‴). ESI–MS: positive mode *m*/*z* 633 [M + Na]^+^; ESI–MS: negative mode *m*/*z* 609 [M − H]^−^.
Compound **7** (peak **2**): Quercetin 3-*O*-β-D-glucopyranosyl-(1 → 6)-*O*-β-D-galactopyranoside, amorphous yellow powder.
^1^H NMR (500 MHz, CD_3_OD) δ_H_ 7.93 (d, *J* = 2.2 Hz, H-2′), 7.65 (dd, *J* = 8.5, 2.2 Hz, H-6′), 6.88 (d, *J* = 8.7 Hz, H-5′), 6.41 (d, *J* = 2.2 Hz, H-8), 6.20 (d, *J* = 2.2 Hz, H-6), 5.14 (d, *J* = 8.3 Hz, H-1″), 4.17 (d, *J* = 8.3 Hz, H-1‴), 3.91 (t, *J* = 3.5 Hz, H-4″), 3.87 (dd, *J* = 8.1, 2.1 Hz, H-6″a), 3.85 (dd, *J* = 9.7, 1.8 Hz, H-2″), 3.80 (t, *J* = 6.1 Hz, H-6‴a), 3.70 (m, H-5″), 3.68 (m, H-6″b), 3.62 (t, *J* = 6.1 Hz, H-6‴b), 3.58 (dd, *J* = 9.8, 3.5 Hz, H-3″), 3.20 (t, *J* = 9.3. Hz, H-4‴), 3.18 (dd, *J* = 5, 1.6 Hz, H-5‴), 3.15 (t, *J* = 9.1 Hz, H-3‴), 3.06 (td, *J* = 7.8, 2.9 Hz, H-2‴). ^13^C NMR (125 MHz, CD_3_OD) δ_C_ 165.7 (C-7), 161.3 (C-5), 157.3 (C-9), 157.0 (C-2), 148.7 (C-4′), 144.6 (C-3′), 134.3 (C-3), 122.5 (C-6′), 121.4 (C-1′), 117.4 (C-2′), 116.1 (C-5′), 104.7 (C-1″), 104.4 (C-10), 103.6 (C-1‴), 99.8 (C-6), 94.5 (C-8), 75.8 (C-5″), 74.7 (C-3″), 74.3 (C-2‴), 72.7 (C-2″), 70.9 (C-4‴), 70.1 (C-4″), 68.6 (C-6″), 61.8 (C-6‴), 77.4 (C-5‴), 77.1 (C-3‴). ESI–MS: positive mode *m*/*z* 649 [M + Na]^+^, 1275 [2M + Na]^+^; ESI–MS: negative mode *m*/*z* 625 [M − H]^−^.

### 3.4. Determination of Total Phenolic Compounds (TPC)

The total phenolic content (TPC) of the *E. dichotomum* extract was determined using the Folin–Ciocalteu method [65]. Due to the high concentration of the extract, a 50-fold dilution in ethanol was performed prior to analysis. An aliquot of 0.5 mL of the diluted extract was mixed with 2.5 mL of 10% (*v*/*v*) Folin–Ciocalteu reagent. After incubation in the dark for 8 min, 2 mL of 7.5% (*w*/*v*) sodium carbonate (Na_2_CO_3_) was added, and the mixture was further incubated in the dark for 1 h. Absorbance was measured at 765 nm using a multi-detection microplate reader (FLUOstar Omega, BMG Labtech, Ortenberg, Germany).

A calibration curve was established using gallic acid standard solutions at five concentrations (10, 50, 75, 100, and 250 µg/g), prepared from a 5 mg/g stock solution in distilled water. The TPC was expressed as milligrams of gallic acid equivalents per gram of dry sample weight (mg GAE/g dry weight).

### 3.5. Determination of Total Flavonoid Content (TFC)

The total flavonoids content of *E. dichotomum* extract was quantified by the aluminum chloride colorimetric method as described by Amzad et al. [66], using quercetin as a standard with some modifications. Initially, 0.5 mL of diluted extract in methanol was mixed with 0.1 mL of aluminum chloride solution (10%), 0.1 mL of potassium acetate (1 M) and 4.3 mL of distilled water. The obtained mixture was firstly incubated at room temperature for 30 min. Then, the absorbance was measured at 415 nm using a multi-detection microplate reader (FLUOstar Omega, BMG Labtech, Germany). A quercetin calibration curve was established to calculate the flavonoid content obtained by preparation of a serial concentration (5 to 60 μg/g). All experiments were performed in triplicate. The results are expressed in mg of quercetin equivalent per gram of dry extract (mg QE/g).

### 3.6. Antioxidant Activity

#### 3.6.1. DPPH Radical Scavenging Capacity

The free radical scavenging activity of *E. dichotomum* extract was evaluated using the 2,2-diphenyl-1-picrylhydrazyl (DPPH) method, according to the protocol described by Wrona et al. [67] with slight modifications. Different concentrations (0.125, 0.25, 0.50, 0.75 and 1 mg/g) of plant extract were prepared in methanol, and 100 µL aliquots were added to 3.5 mL of a freshly prepared DPPH solution (30 µg/g) in methanol; Sigma-Aldrich, Steinheim, Germany). A DPPH solution without extract was used as a blank. All samples were incubated in the dark for 15 min. The absorbance was then measured at 515 nm, against methanol, using a multi-detection microplate reader (FLUOstar Omega, BMG Labtech, Germany). The DPPH solution was prepared daily and its concentration was verified using a calibration curve obtained from serial concentrations of DPPH (4, 8, 16, 32, and 64 µg/g).

The antioxidant activity was expressed as the percentage of DPPH inhibition (I%), calculated according to Equation (1):

(1) $$I\%=\frac{A_{0}-A}{A_{0}}\times 100$$

where A_0_ represents the absorbance of the control (DPPH in methanol) and A the absorbance of the sample containing the extract. The percentage inhibition values obtained after 30 min were plotted against the extract concentrations to establish the antioxidant activity curve. The IC_50_ value, defined as the concentration of extract required to inhibit 50% of DPPH radicals, was determined by linear regression. A low IC_50_ value indicates high antioxidant activity.

#### 3.6.2. Ferric Reducing Antioxidant Power (FRAP) Assay

The reducing power of iron in *E. dichotomum* extract was evaluated according to the method described by Bouabid et al. [68], with slight modifications. Aliquots of 1 mL of different dilutions of the extract prepared in methanol were mixed with 2.5 mL of phosphate buffer (0.2 M; pH 6.6) and 2.5 mL of potassium ferricyanide (1%) (Sigma-Aldrich, St. Louis, MO, USA). The reaction mixture was then incubated at 50 °C for 20 min. After incubation, 2.5 mL of trichloroacetic acid (10%) was added to stop the reaction, and the samples were centrifuged at 3000 rpm for 10 min.

A volume of 2.5 mL of the supernatant obtained was mixed with 2.5 mL of distilled water and 0.5 mL of ferric chloride (0.1%) and then homogenized vigorously. The absorbance was measured at 700 nm using a multi-detection microplate reader (FLUOstar Omega, BMG Labtech, Germany), UV–Visible spectrophotometer (UV-1700, Shimadzu, Tokyo, Japan), against a blank prepared under the same conditions by replacing the plant ex-tract with methanol.

A calibration curve was established using ascorbic acid (Merck, Darmstadt, Germany) in a range of serial concentrations (0.001, 0.025, 0.05, 0.1, and 0.2 mg/g). The results were expressed in milligrams of ascorbic acid equivalent per gram of extract (mg AAE/g extract).

#### 3.6.3. ABTS (2,2′,-Azino-bis(3-ethylbenzothiazoline-6-sulfonic acid) Assay

The ABTS radical scavenging activity of *E. dichotumum* extract was evaluated according to the method described by Peng et al. [69]. First, 1 mL of ABTS stock solution (7 mM) (Sigma-Aldrich, Steinheim, Germany) was mixed with 88 µL of potassium persulfate solution (140 mM). The mixture was then incubated at room temperature, protected from light, overnight to generate the ABTS^+^ radical.

The resulting solution was then diluted with phosphate-buffered saline (0.2 M; pH 7.4) until an absorbance of 0.70 ± 0.02 at 734 nm was obtained. A volume of 3 mL of this ABTS solution was mixed with 1 mL of the extract at different concentrations (25, 50, 75, 100, and 250 µg/mL). The reaction was carried out in the dark for 6 min, then the absorbance was measured at 734 nm using a multi-detection microplate reader (FLUOstar Omega, BMG Labtech, Germany).

Samples containing the same volume of methanol and the same dilution of ascorbic acid were used as blank and positive controls, respectively. The ABTS radical scavenging capacity was calculated according to Equation (2):

(2) $$ABTS\;radical\;scavenging\;activity\left(\%\right)=1-\frac{A_{1}-A_{2}}{A_{0}}\times 100$$

where A_1_ corresponds to the absorbance of the ABTS-sample mixture, A_2_ to that of the solvent-sample mixture, and A_0_ to the absorbance of the solvent-ABTS mixture.

### 3.7. Antitumoral Activity

#### 3.7.1. Cell Culture and Reagents

The A375 human amelanotic melanoma cell line was acquired from the European Collection of Authenticated Cell Cultures (ECACC 88113005). The HaCaT cell line, consisting of immortalized non-tumorigenic human keratinocytes, was supplied by CLS Cell Lines Services (Eppelheim, Germany). Both A375 and HaCaT cell lines were cultured in High-Glucose Dulbecco’s Modified Eagle’s Medium (DMEM, PAN-Biotech, Aidenbach, Germany) supplemented with 10% fetal bovine serum (FBS, PAN-Biotech, Aidenbach, Germany), 2 mM L-glutamine, 1% pen/strep (100 U/mL penicillin, 100 ug/mL streptomycin and 1% fungizone (Grisp, Porto, Portugal), and kept 37 °C under 5% CO_2_ conditions.

#### 3.7.2. Assessment of Cell Viability

A375 and HaCaT cells were plated out in 96-well plates at a density of 3.5 × 10^4^ (A375) and 6.0 × 10^4^ (HaCaT). After allowing sufficient adhesion time (24 h) at 37 °C under 5% CO_2_, cells were treated with falcarinol at a concentration of 0.25, 0.5, 1, 2, 4, 8 µM for 24 h., They were subsequently evaluated to determine cell viability.

Cell viability was evaluated according to Twentyman and Luscombe [70] using the colorimetric MTT [3-(4,5-dimethyl-2-thiazolyl)-2,5-diphenyl tetrazolium bromide] assay (purity ≥ 98%; Sigma-Aldrich, St. Louis, MO, USA). After 24 h exposure, 50 µL of MTT solution (1.0 mg/mL in PBS) was added to each well and incubated for 4 h at 37 °C. The medium was then removed and replaced with 150 µL of DMSO in order to solubilize the formazan crystals and the plates were agitated in the dark at room temperature for 2 h. Absorbance readings were taken at 570 nm with a Synergy HT^®^ Multi-Mode microplate reader (BioTek^®^, Winooski, VT, USA). Cells treated with vehicle (DMSO) only served as the negative control. The IC_20_ and IC_50_ corresponding to a 20% and 50% reduction in viability, respectively, were then determined for Falcarinol.

### 3.8. Statistical Analysis

For the determinations of Total Phenolic Content (TPC), Total Flavonoid Content (TFC), and antioxidant activity, each sample was analyzed three times. The data presented represent the means of the results obtained, with errors expressed as standard deviations. Student’s *t*-test was conducted to differentiate results within the same group. Cell viability values are presented as the mean ± standard deviation (SD) from at least three replicates of three independent assays. Statistical comparisons were performed using one-way ANOVA in Sigma Plot 14.0 (Systat Software Inc., San Jose, CA, USA). Post hoc analyses were carried out using Dunnett’s (parametric) test: *p*-values < 0.05 or *p* < 0.001 is considered statistically significant.

## 4. Conclusions

The present investigation described a comparative phytochemical profile via UHPLC–MS, in combination with a phytochemical examination of the diethyl ether (Et_2_O) extract derived from the aerial parts of *Eryngium dichotomum*, a species that has remained largely unexplored in the literature. The LC–MS profile showed a diverse array of chemical compounds, including phenolic acids, flavonoids, triterpene saponins, hydroxylated fatty acids, and polyacetylenes, tentatively assigned. These findings are in agreement with previous phytochemical studies conducted on *Eryngium* species, which highlighted the occurrence of flavonoids and polyacetylene derivatives as characteristic constituents of the genus. Interestingly, seven compounds were isolated after chromatography and fully characterized by NMR (^1^H, ^13^C, COSY, HSQC, and HMBC) and mass spectrometry. Among these metabolites, three compounds, two oxylipins and one C17-polyacetylene-type falcarinol derivative, are reported herein for the first time in the genus *Eryngium*.

In addition, two glycosylated flavonoids identified as quercetin and kaempferol derivatives are reported for the first time in this species, further highlighting the chemical diversity and chemotaxonomic significance of *Eryngium* species.

Furthermore, the isolated falcarinol was evaluated for its potential antitumor activity against melanoma A375 cancer cells and non-tumorigenic HaCaT keratinocyte cells. The results demonstrated a pronounced decrease in the viability of A375 melanoma cells. In contrast, HaCaT keratinocytes exhibited markedly higher resistance to the compound. These findings indicate that falcarinol shows higher cytotoxicity toward melanoma cells than non-tumorigenic cells, with an approximately 8-fold difference in sensitivity between A375 and HaCaT cells.

The antioxidant properties of the extracts were also investigated by quantifying their total phenolic (TPC) and total flavonoid (TFC) contents, and by evaluating their radical scavenging and reducing capacities using the DPPH, ABTS, and ferric reducing antioxidant power (FRAP) assays. The *n*-butanol (BuOH) extract demonstrated higher antioxidant activity in the DPPH and ABTS assays than the diethyl ether (Et_2_O) extract, which showed a significant FRAP value.

The outcomes of this study reveal that *E. dichotomum* holds considerable promise as a source of polyacetylenes endowed with anticancer and antioxidant properties. To fully realize its therapeutic potential, further research should focus on the detailed isolation and purification of additional polyacetylenes and antioxidant compounds, thereby enabling the comprehensive evaluation of their biological activities.

## Figures and Tables

**Table 2:** Total phenolic and flavonoid contents of *E. dichotomum* extracts compared to related species.

	Extracts	TPC (mg GAE/g) *	TFC (mg QE/g) **
*E. dichotomum* (this study)	Et_2_O	194.89 ± 0.56 ^a^	96.80 ± 0.35 ^a^
	BuOH	366.24 ± 0.93 ^b^	231.17 ± 0.16 ^b^
*E. campestre* [42]	EtOAc H_2_O	27.77 -	- 7.54
*E. caucasicum* [43]	MeOH	62.3	25.3
*E. kotschyi* [44]	EtOAc	173.71	-
*E. billardieri* [45]	MeOH/H_2_O	19.25	

* GAE: gallic acid equivalents; ** QE: quercetin equivalents. ^a,b^: different letters in a column indicate significantly different results according to the *t*-test (*p* < 0.05).

**Table 3:** Antioxidant activity of *Eryngium dichotomum* extracts.

Samples	DPPH (IC_50_ µg/g) **	FRAP (mg AA/g) *	ABTS (IC_50_ µg/g) **
Et_2_O	21.23 ± 0.36 ^a^	164.11 ± 1.01 ^a^	119.19 ± 1.01 ^a^
BuOH	18.36 ± 0.21 ^b^	27.69 ± 0.54 ^b^	94.15 ± 0.69 ^b^
Trolox	11.97 ± 0.41	-	23.16 ± 0.54
Ascorbic acid	3.36 ± 0.13	-	-

* AA: Ascorbic Acid; ** IC: Inhibition Concentration. ^a,b^: different letters in a column indicate significantly different results according to the *t*-test (*p* < 0.05).

**Table 4:** IC_50_ values (µM) for Falcarinol in A375 melanoma cells and HaCaT keratinocytes after 24 h treatment.

Cell Line	A375	HaCaT
IC_50_	1.10 µM	9.03 µM

## Data Availability

The original contributions presented in this study are included in the article/Supplementary Material. Further inquiries can be directed to the corresponding authors.

## Supplementary Materials

The following supporting information can be downloaded at: https://www.mdpi.com/article/10.3390/molecules31111959/s1, Spectroscopic data (NMR and Mass spectrum of the compounds (**1**–**7**)).

## References

[1] Wörz A. (1999). A Taxonomic Index of the Species of *Eryngium* L. (Apiaceae: Saniculoideae). Stuttg. Beitr. Naturk. Ser. A.

[2] Wang P., Su Z., Yuan W., Deng G., Li S. (2012). Phytochemical Constituents and Pharmacological Activities of *Eryngium* L. (Apiaceae). Pharm. Crops.

[3] Christensen L.P. (2011). Aliphatic C17-Polyacetylenes of the Falcarinol Type as Potential Health Promoting Compounds in Food Plants of the Apiaceae Family. Recent Pat. Food Nutr. Agric..

[4] Park J., Jang S., Lee S.O., Park I.W., Li S., Nam A.Y., Shim J.H., Na M.K. (2025). Cytotoxic Activity of C17 Polyacetylenes from the Roots of *Glehnia littoralis* against Drug-Resistant Colorectal and Lung Cancer Cells. J. Nat. Med..

[5] Stefanson A.L., Bakovic M. (2018). Falcarinol Is a Potent Inducer of Heme Oxygenase-1 and Was More Effective than Sulforaphane in Attenuating Intestinal Inflammation at Diet-Achievable Doses. Oxid. Med. Cell. Longev..

[6] Quézel P., Santa S. (1962). Nouvelle Flore de l′Algérie et Des Régions Désertiques Méridionales.

[7] Bouzergoune F., Ciavatta M.L., Bitam F., Carbone M., Aberkane M.C., Gavagnin M. (2016). Phytochemical study of *Eryngium triquetrum*: Isolation of polyacetylenes and lignans. Planta Med..

[8] Djebara A., Ciavatta M.L., Mathieu V., Colin M., Bitam F., Carbone M., Gavagnin M. (2019). Oxygenated C17 polyacetylene metabolites from Algerian *Eryngium tricuspidatum* L. roots: Structure and biological activity. Fitoterapia.

[9] Medbouhi A., Benbelaïd F., Djabou N., Beaufay C., Bendahou M., Quetin-Leclercq J., Muselli A. (2019). Essential oil of Algerian *Eryngium campestre*: Chemical variability and evaluation of biological activities. Molecules.

[10] Medbouhi A., Tintaru A., Beaufay C., Naubron J.V., Djabou N., Costa J., Muselli A. (2018). Structural elucidation and cytotoxicity of a new 17-membered ring lactone from Algerian *Eryngium campestre*. Molecules.

[11] Nacef S., Msaddek M., Ben Jannet H., Attia S., Chriaa J., Bakhrouf A., Mighri Z.J. (2003). Isolation and structural elucidation of a steroid and a heteroside from the plant *Eryngium dichotomum*. GC/MS identification of some plants and study of their antibacterial activities. Soc. Alger. Chim..

[12] Nacef S., Ben Jannet H., Hamza M.A., Mighri Z. (2008). Contribution to the phytochemical investigation of the plant *Eryngium dichotomum* Desf. (Apiaceae) from Tunisia. J. Soc. Chim. Tunis..

[13] Landoulsi A., Hennebelle T., Bero J., Rivière C., Sahpaz S., Quetin-Leclercq J., Neut C., Benhamida J., Roumy V. (2020). Antimicrobial and light-enhanced antimicrobial activities, cytotoxicity and chemical variability of all Tunisian *Eryngium* species. Chem. Biodivers..

[14] Zengin G., Yagi S., Eldahshan O.A., Singab A.N., Selvi S., Rodrigues M.J., Custodio L., Dall’Acqua S., Ponnaiya S.K.M., Aly S.H. (2024). Decoding chemical profiles and biological activities of aerial parts and roots of *Eryngium thorifolium* Boiss by HPLC-MS/MS, GC-MS and in vitro chemical assays. Food Biosci..

[15] Kikowska M., Kruszka D., Derda M., Hadaś E., Thiem B. (2020). Phytochemical screening and acanthamoebic activity of shoots from in vitro cultures and in vivo plants of *Eryngium alpinum* L.—The endangered and protected species. Molecules.

[16] Ozarowski M., Thiem B., Mikolajczak P.L., Piasecka A., Kachlicki P., Szulc M., Kaminska E., Bogacz A., Kujawski R., Bartkowiak-Wieczorek J. (2015). Improvement in Long-Term Memory following Chronic Administration of *Eryngium planum* Root Extract in Scopolamine Model: Behavioral and Molecular Study. Evid. Based Complement. Altern. Med..

[17] Vuković N.L., Vukić M.D., Đelić G.T., Kacaniova M.M., Cvijović M. (2018). The investigation of bioactive secondary metabolites of the methanol extract of *Eryngium amethystinum*. Kragujev. J. Sci..

[18] Zarnack J., Hildebrandt B., Hiller K., Otto A. (1979). To the knowledge of the compounds contained in some Saniculoideae. part XXXIII. isolation of flavonol glycosides from *Eryngium giganteum* M. B. Z. Chem..

[19] Hiller K., Otto A., Gruendemann E. (1980). Isolation of kaempferol-3-*O*-(6-*O*-β-D-glucopyranosyl)-β-D-galactopyranoside, a new flavonol glycoside from *Eryngium planum* L. Part 34: Knowledge of the constituents of some Saniculoideae. Pharmazie.

[20] Leokadia S.P. (1983). Kaempferol 3,7-Dirhamnoside from *Eryngium planum* L.. Z. Chem..

[21] Zarnack J., Hiller K., Otto A. (1977). The Components of Several Saniculoideae. XXVIII. Isolation of Kaempferol 3,7-di-*O*-rhamnoside from *Eryngium planum* L.. Z. Chem..

[22] Ikramov M.T., Bandyukova V.A., Khalmatov K.K. (1971). Flavonoids of some *Eryngium* species. Khim. Prir. Soedin..

[23] Kartnig T., Wolf J. (1993). Flavonoids from the aerial parts of *Eryngium campestre*. Planta Med..

[24] Zhang Z.Z., Li S.Y., Ownby S., Wang P., Yuan W., Zhang W.L., Beasley R.S. (2008). Phenolic compounds and rare polyhydroxylated triterpenoid saponins from *Eryngium yuccifolium*. Phytochemistry.

[25] Hohmann J., Páll Z., Günther G., Máthé I. (1997). Flavonolacyl glycosides of the aerial parts of *Eryngium campestre*. Planta Med..

[26] Kartal M., Mitaine-Offer A.C., Paululat T., Abu-Asaker M., Wagner H., Mirjolet J.F., Guilbaud N., Lacaille-Dubois M.A. (2006). Triterpene saponins from *Eryngium campestre*. J. Nat. Prod..

[27] Li Z., Tran V.H., Duke R.K., Ng M.C., Yang D., Duke C.C. (2009). Synthesis and biological activity of hydroxylated derivatives of linoleic acid and conjugated linoleic acids. Chem. Phys. Lipids.

[28] Zha S., Kuwano K., Shibahara T., Ishibashi F. (2020). Algicidal hydroxylated C18 unsaturated fatty acids from the red alga *Tricleocarpa jejuensis*: Identification, synthesis and biological activity. Fitoterapia.

[29] Matsuura H., Saxena G., Farmer S.W., Hancock R.E.W., Towers G.H.N. (1996). Antibacterial and antifungal polyine compounds from *Glehnia littoralis* ssp. *leiocarpa*. Planta Med..

[30] Ayoub N., Al-Azizi M., Konig W., Kubeczka K.H. (2006). Essential oils and a novel polyacetylene from *Eryngium yuccifolium* Michx. (Apiaceae). Flavour Fragr. J..

[31] Joergen L., Lars P.C., Tove T. (1992). Acetylenes from roots of *Eryngium bourgatii*. Phytochemistry.

[32] Ghandi M., Mostashari A., Karegar M., Barzegar M. (2007). Efficient synthesis of α-monoglycerides via solventless condensation of fatty acids with glycerol carbonate. J. Am. Oil Chem. Soc..

[33] Kikowska M., Chanaj-Kaczmarek J., Derda M., Budzianowska A., Thiem B., Ekiert H., Szopa A. (2022). The evaluation of phenolic acids and flavonoids content and antiprotozoal activity of *Eryngium* species Biomass produced by Biotechnological Methods. Molecules.

[34] Cádiz-Gurrea M.L., Fernández-Arroyo S., Joven J., Segura-Carretero A. (2013). Comprehensive characterization by UHPLC-ESI-Q-TOF-MS from an *Eryngium bourgatii* extract and their antioxidant and anti-inflammatory activities. Food Res. Int..

[35] Kikowska M., Thiem B., Szopa A., Klimek-Szczykutowicz M., Rewers M., Sliwinska E., Ekiert H. (2019). Comparative analysis of phenolic acids and flavonoids in shoot cultures of *Eryngium alpinum* L.: An endangered and protected species with medicinal value. Plant Cell Tissue Organ Cult..

[36] Wang P., Yuan W., Deng G., Su Z., Li S. (2013). Triterpenoid saponins from *Eryngium yuccifolium* ‘Kershaw Blue’. Phytochem. Lett..

[37] Ko Y.-C., Choi H.S., Kim J.-H., Kim S.-L., Yun B.-S., Lee D.-S. (2020). Coriolic Acid (13-(S)-Hydroxy-9Z, 11E-octadecadienoic Acid) from Glasswort (*Salicornia herbacea* L.) Suppresses Breast Cancer Stem Cell through the Regulation of c-Myc. Molecules.

[38] Ngo-Duy C.C., Destaillats F., Keskitalo M., Arul J., Angers P. (2009). Triacylglycerols of Apiaceae seed oils: Composition and regiodistribution of fatty acids. Eur. J. Lipid Sci. Technol..

[39] Avato P., Fanizzi F.P., Rosito I. (2001). The genus *Thapsia* as a source of petroselinic acid. Lipids.

[40] Benmerache A., Magid A.A., Berrehal D., Kabouche A., Voutquenne-Nazabadioko L., Messaili S., Kabouche Z. (2016). Chemical composition, antibacterial, antioxidant and tyrosinase inhibitory activities of glycosides from aerial parts of *Eryngium tricuspidatum* L.. Phytochem. Lett..

[41] Hatami M., Karimi M., Aghaee A., Bovand F., Ghorbanpour M. (2022). Morphological diversity, phenolic acids, and antioxidant properties in eryngo (*Eryngium caucasicum* Trautv): Selection of superior populations for agri-food industry. Food Sci. Nutr..

[42] Bouzidi S., Benkiki N., Hachemi M., Haba H. (2017). Investigation of in vitro antioxidant activity and in vivo antipyretic and anti-inflammatory activities of Algerian *Eryngium campestre* L.. Curr. Bioact. Compd..

[43] Nabavi S.M., Ebrahimzadeh M.A., Nabavi S.F., Jafari M. (2008). Free radical scavenging activity and antioxidant capacity of *Eryngium caucasicum* Trautv and *Froripia subpinnata*. Pharmacologyonline.

[44] Paşayeva L., Şafak E.K., Arıgün T., Fatullayev H., Tugay O. (2020). In vitro antioxidant capacity and phytochemical characterization of *Eryngium kotschyi* Boiss. J. Pharm. Pharmacogn. Res..

[45] Roudbari M., Barzegar M., Sendra E., Casanova-Martínez I., Rodríguez-Estrada M., Carbonell-Barrachina Á.A. (2025). Characterization of the different chemical components and nutritional properties of two *Eryngium* species. Foods.

[46] Marčetić M.D., Petrović S.D., Milenković M.T., Niketić M.S. (2014). Composition, antimicrobial and antioxidant activity of the extracts of *Eryngium palmatum* Pančić and Vis. (*Apiaceae*). Cent. Eur. J. Biol..

[47] Ebrahimzadeh M.A., Nabavi S.F., Nabavi S.M. (2009). Antioxidant activity of leaves and inflorescence of *Eryngium caucasicum* Trautv at flowering stage. Pharmacogn. Res..

[48] Kaurinovic B., Vastag D., Shahidi F. (2021). Flavonoids and phenolic acids as potential natural antioxidants in food and health. Handbook of Antioxidants for Food Preservation.

[49] Zhang Y., Cai P., Cheng G., Zhang Y. (2022). A Brief Review of Phenolic Compounds Identified from Plants: Their Extraction, Analysis, and Biological Activity. Nat. Prod. Commun..

[50] Ouamnina A., Alahyane A., Elateri I., Boutasknit A., Abderrazik M. (2024). Relationship between Phenolic Compounds and Antioxidant Activity of Some Moroccan Date Palm Fruit Varieties (*Phoenix dactylifera* L.): A Two-Year Study. Plants.

[51] Jan R., Khan M., Asaf S., Lubna, Asif S., Kim K.-M. (2022). Bioactivity and Therapeutic Potential of Kaempferol and Quercetin: New Insights for Plant and Human Health. Plants.

[52] Lesjak M., Beara I., Simin N., Pintać D., Majkić T., Bekvalac K., Orčić D., Mimica-Dukić N. (2018). Antioxidant and anti-inflammatory activities of quercetin and its derivatives. J. Funct. Foods.

[53] Grabowska K., Pietrzak W., Paśko P., Sołtys A., Galanty A., Żmudzki P., Nowak R., Podolak I. (2023). Antihyaluronidase and Antioxidant Potential of Atriplex sagittata Borkh. in Relation to Phenolic Compounds and Triterpene Saponins. Molecules.

[54] Biswas T., Dwivedi U.N. (2019). Plant triterpenoid saponins: Biosynthesis, in vitro production, and pharmacological relevance. Protoplasma.

[55] Kostić K., Brborić J., Delogu G., Simić M.R., Samardžić S., Maksimović Z., Dettori M.A., Fabbri D., Kotur-Stevuljević J., Saso L. (2023). Antioxidant Activity of Natural Phenols and Derived Hydroxylated Biphenyls. Molecules.

[56] Xie Q., Wang C. (2022). Polyacetylenes in herbal medicine: A comprehensive review of its occurrence, pharmacology, toxicology, and pharmacokinetics (2014–2021). Phytochemistry.

[57] Kobaek-Larsen M., Rime B., El-Houri R.B., Christensen L.P., Al-Najami I., Fretté X., Baatrup G. (2017). Dietary polyacetylenes, falcarinol and falcarindiol, isolated from carrots prevents the formation of neoplastic lesions in the colon of azoxymethane- induced rats. Food Funct..

[58] Cheung S.S.C., Hasman D., Khelifi D., Tai J., Smith R.W., Warnock G.L. (2019). Devil’s Club Falcarinol-Type Polyacetylenes Inhibit Pancreatic Cancer Cell Proliferation. Nutr. Cancer.

[59] Zaini R., Brandt K., Clench M., Maitre C. (2012). Effects of bioactive compounds from carrots (*Daucus carota* L.), polyacetylenes, beta-carotene and lutein on human lymphoid leukaemia cells. Anti-Cancer Agents Med. Chem..

[60] Cheung S.S., Khelifi D., Yang Z.S., Hasman D., Dai L.J., Xin J.X., Wang B., Tai J. (2023). Devil’s club falcarinol inhibits human glioblastoma cell proliferation and tumor progression in xenografts. Pharmacol. Res. Mod. Chin. Med..

[61] Zidorn C., Jöhrer K., Ganzera M., Schubert B., Sigmund E.M., Mader J., Ellmerer E.P., Stuppner H. (2005). Polyacetylenes from the Apiaceae vegetables carrot, celery, fennel, parsley, and parsnip and their cytotoxic activities. J. Agric. Food Chem..

[62] Christensen L.P. (2020). Bioactive C17 and C18 acetylenic oxylipins from terrestrial plants as potential lead compounds for anticancer drug development. Molecules.

[63] Resetar M., Liu X., Herdlinger S., Kunert O., Pferschy-Wenzig E.M., Latkolik S., Steinacher T., Schuster D., Bauer R., Dirsch V.M. (2020). Polyacetylenes from *Oplopanax horridus* and Panax ginseng: Relationship between Structure and PPARγ Activation. J. Nat. Prod..

[64] Jin H.R., Liao Y., Li X., Zhang Z., Zhao J., Wang C.Z., Huang W.H., Li S.P., Yuan C.S., Du W. (2014). Anticancer compound Oplopantriol A kills cancer cells through inducing ER stress and BH3 proteins Bim and Noxa. Cell Death Dis..

[65] Song X.C., Canellas E., Asensio E., Nerín C. (2020). Predicting the Antioxidant Capacity and Total Phenolic Content of Bearberry Leaves by Data Fusion of UV–Vis Spectroscopy and UHPLC/Q-TOF-MS. Talanta.

[66] Amzad Hossain M., Shah M.D. (2015). A Study on the Total Phenols Content and Antioxidant Activity of Essential Oil and Different Solvent Extracts of Endemic Plant *Merremia borneensis*. Arab. J. Chem..

[67] Wrona M., Nerín C., Alfonso M.J., Caballero M.Á. (2017). Antioxidant packaging with encapsulated green tea for fresh minced meat. Innov. Food Sci. Emerg. Technol..

[68] Bouabid K., Lamchouri F., Toufik H., Faouzi M.E.A. (2020). Phytochemical investigation, in vitro and in vivo antioxidant properties of aqueous and organic extracts of toxic plant: *Atractylis gummifera* L.. J. Ethnopharmacol..

[69] Peng Q., Huang Z., Liang G., Bi Y., Kong F., Wang Z., Tan S., Zhang J. (2024). Preparation of protein-stabilized *Litsea cubeba* essential oil nano-emulsion by ultrasonication: Bioactivity, stability, in vitro digestion, and safety evaluation. Ultrason. Sonochem..

[70] Twentyman P., Luscombe M. (1987). A Study of Some Variables in a Tetrazolium Dye (MTT) Based Assay for Cell Growth and Chemosensitivity. Br. J. Cancer.

