# Supplementary material for: Exploring the Chemical Profile and Biological Activities of Eryngium dichotomum: UHPLC–MS/NMR Characterization, and In Vitro Antioxidant Activity Along with the Antitumor Effect of Falcarinol
Source: Molecules. 2026 Jun 4;31(11):1959. doi: 10.3390/molecules31111959 (PMC13258210; doi:10.3390/molecules31111959)
Supplement: Supplementary file 1 [file molecules-31-01959-s001.zip › molecules-4286637-supplementary.pdf]

Supplementary Material

Exploring the chemical profile and biological activities of *Eryngium dichotomum*: UHPLC–MS/NMR characterization, and *in vitro* antioxidant activity along with the antitumor effect of falcarinol

Roufia Mezaache [1,2], Habiba Laraoui [2,3], Anis Bertella [4,5], Verónica Bastos [6], Helena Oliveira [6], Patrick Pale [7], Aurelien Blanc [7], Stefan Chassaing [7], Fatma Bitam [1,2,*], Oana - Crina Bujor [8], Diana C.G.A Pinto [9], Liliana Bădulescu [8], Artur M.S. Silva [9].

[1] Department of Pharmacy, Faculty of Medicine, University Batna 2, Batna 05000, Algeria

[2] Laboratory of Chemistry and Environmental Chemistry (L.C.C.E), Department of Chemistry, Faculty of Science, University of Batna 1, Batna 05000, Algeria ; f.bitam@univ-batna2.dz; r.mezaache@univ-batna2.dz

[3] Department of Matter Sciences, Faculty of Exact Sciences, University of Biskra, Biskra 07000, Algeria; habiba.laraoui@univ-biskra.dz

[4] Laboratory of Applied Molecular Biology (LAMB), Faculty of Life and Nature Sciences, Abbes Laghrour University Khenchela, BP 1252 Road of Batna, Khenchela 40004, Algeria.

[5] Food Science Laboratory (LSA), Department of Food Engineering, Institute of Veterinary and Agricultural Sciences, University Batna 1-Hadj Lakhdar, Batna 05000, Algeria; anis.bertella@univ-khenchela.dz

[6] Department of Biology & CESAM, University of Aveiro, Aveiro, Portugal; holiveira@ua.pt

[7] Laboratory of organometallic catalysis, organic synthesis and health (COSyS), Institut de Chimie UMR 7177, University of Strasbourg, Strasbourg, France; ppale@unistra.fr, ablanc@unistra.fr, chassaing@unistra.fr.

[8] Research Center for Studies of Food and Agricultural Products Quality, University of Agronomic Sciences and Veterinary Medicine of Bucharest, 59, Mărăști Blvd., Bucharest, 011464, Romania; oana.bujor@qlab.usamv.ro ; liliana.badulescu@qlab.usamv.ro

[9] LAQV-REQUIMTE, Department of Chemistry, University of Aveiro, Campus de Santiago, 3810-193 Aveiro, Portugal; diana@ua.pt

* Correspondence: f.bitam@univ-batna2.dz

S1. ^{1}H NMR spectrum of compound **1** (500 MHz, $CDCl_3$)
S2. COSY spectrum of compound **1** (500 MHz, $CDCl_3$)
S3. ESIMS Mass spectrum of compound **1**
S4. ESIMS spectrum of compound **1** (500 MHz, $CDCl_3$)
S5. ^{1}H NMR spectrum of compound **2** (500 MHz, $CDCl_3$)
S6. ESIMS spectrum of compound **2**
S7. ^{1}H NMR spectrum of compound **3** (500 MHz, $CDCl_3$)
S8. ^{13}CNMR spectrum of compound **3** (125 MHz, $CDCl_3$)
S9. HSQC spectrum of compound **3** (500 MHz, $CDCl_3$)
S10. HMBC spectrum of compound **3** (500 MHz, $CDCl_3$)
S11. ESIMS spectrum of compound **3**
S12. ESIMS spectrum of compound **3**
S13. ^{1}H NMR spectrum of compound **4** (500 MHz, $CDCl_3$)
S14. COSY spectrum of compound **4** (500 MHz, $CDCl_3$)
S15. ESIMS spectrum of compound **4**
S16. ^{1}H NMR spectrum of compound **5**
S17. ESIMS spectrum of compound **5**
S18. ESIMS spectrum of compound **5**
S19. ^{1}H NMR spectrum of compound **6** (500 MHz, CD_3OD)
S20. COSY spectrum of compound **6** (500 MHz, CD_3OD)
S21. HSQC spectrum of compound **6** (500 MHz, CD_3OD)
S22. HMBC spectrum of compound **6** (500 MHz, CD_3OD)
S23. ESIMS Mass spectrum of compound **6**
S24. ESIMS Mass spectrum of compound **6**
S25. ^{1}H NMR spectrum of compound **7** (500 MHz, CD_3OD)
S26. COSY spectrum of compound **7** (500 MHz, CD_3OD)
S27. HSQC spectrum of compound **7** (500 MHz, CD_3OD)
S28. HMBC spectrum of compound **7** (500 MHz, CD_3OD)
S29. ESIMS Mass spectrum of compound **7**
S30. ESIMS Mass spectrum of compound **7**

Compound 1: *(9*Z*,11*E*)*-13-hydroxyoctadeca-9,11-dienoic acid

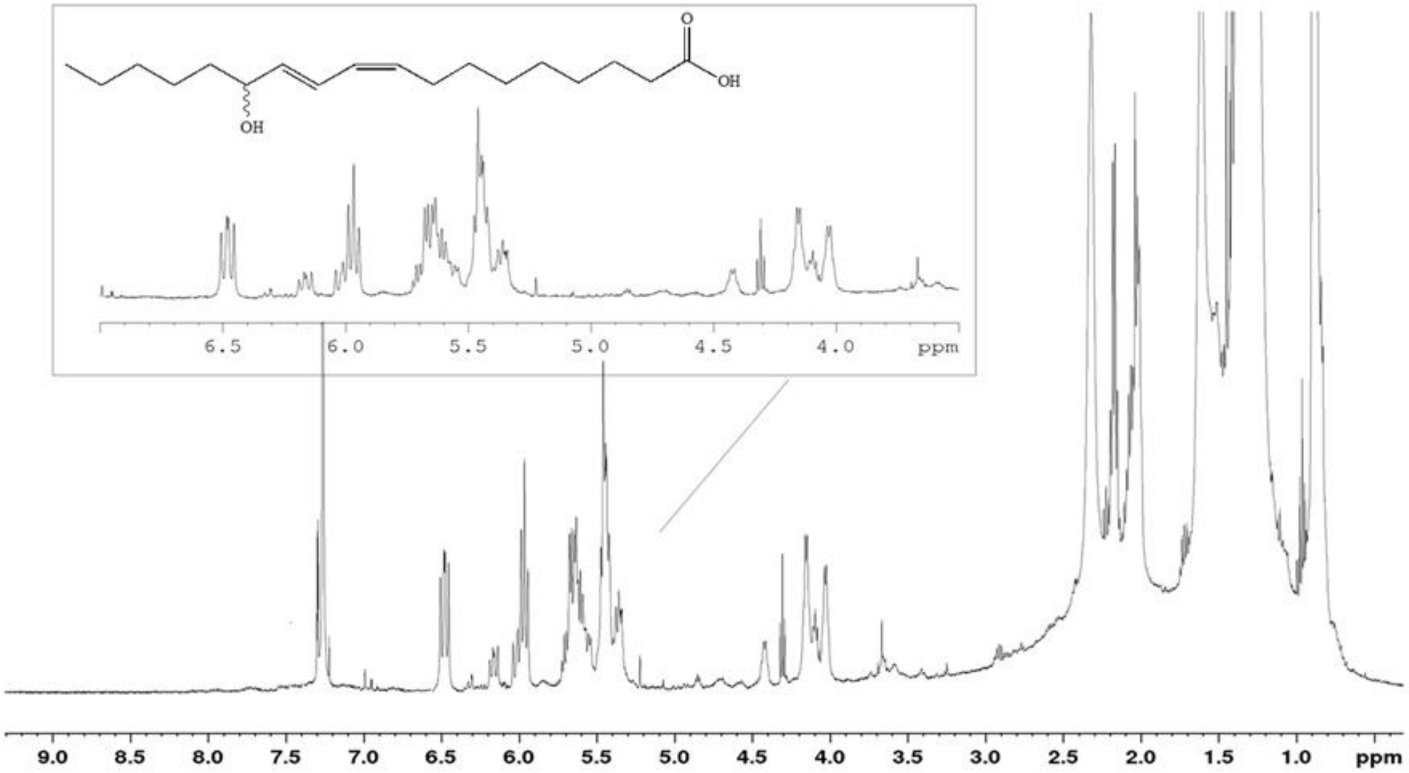

S1. ^{1}H NMR spectrum of compound **1** (500 MHz, $CDCl_3$)

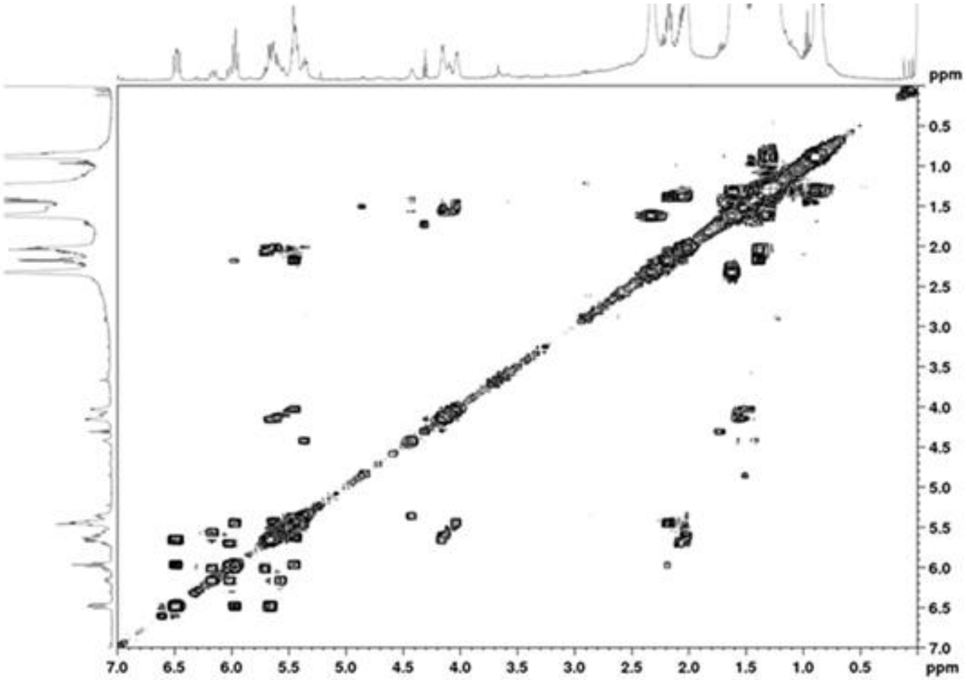

S2. COSY spectrum of compound **1** (500 MHz, $CDCl_3$)

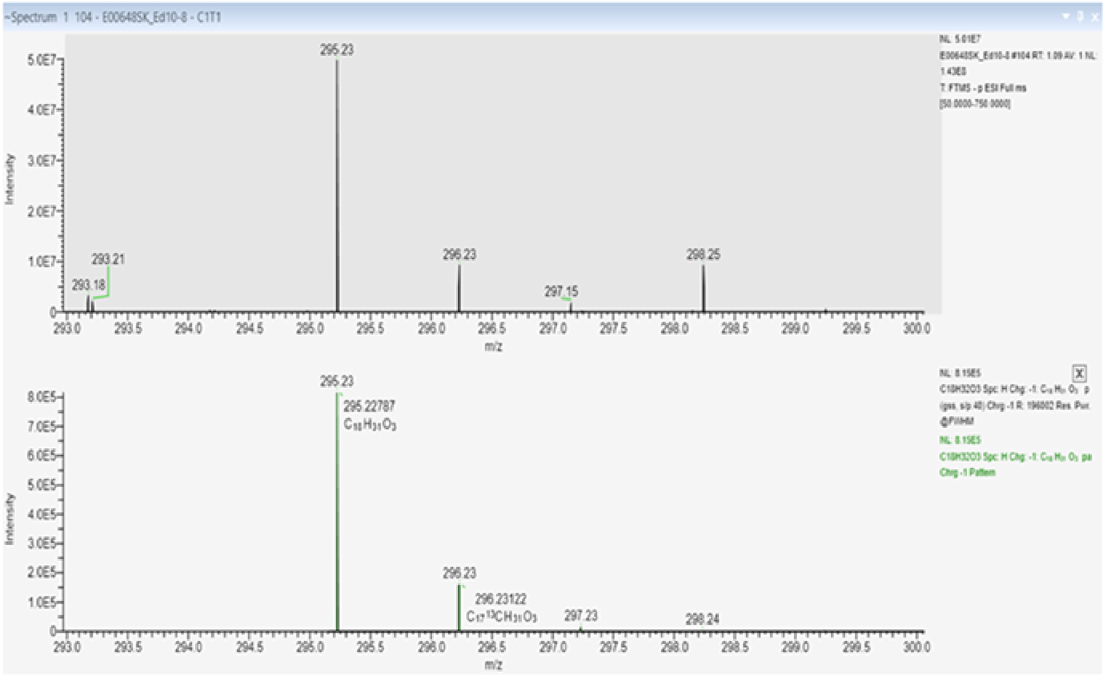

S3. ESIMS spectrum of compound **1**

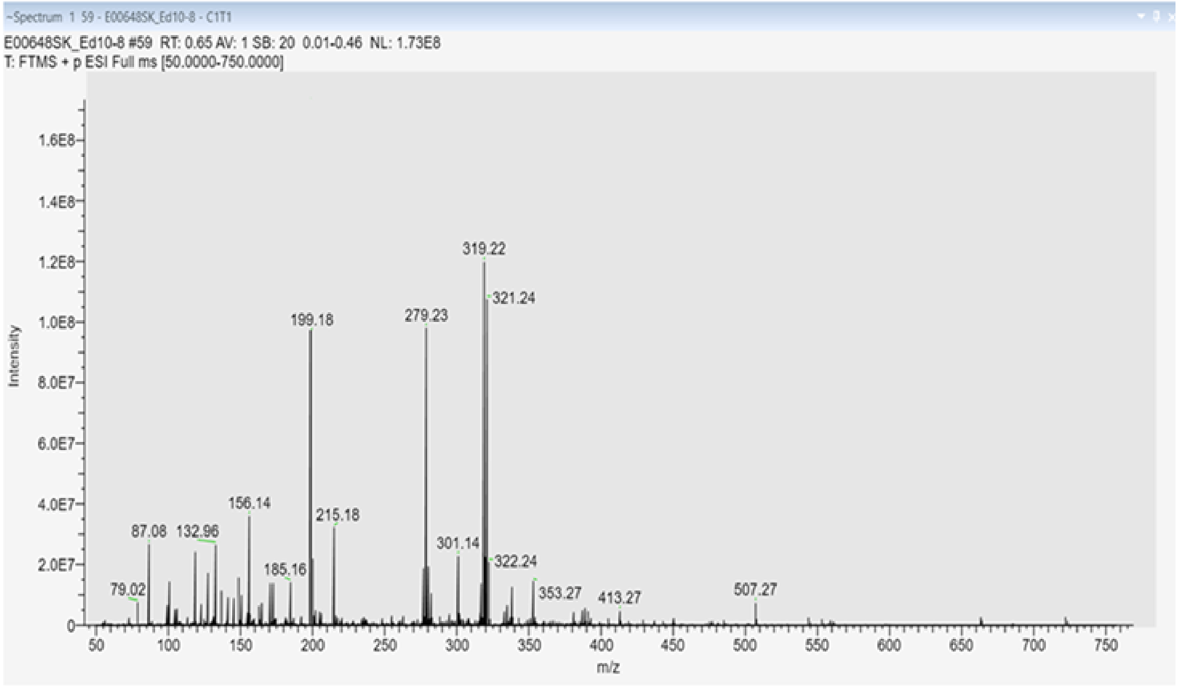

S4. ESIMS spectrum of compound **1**

Compound 2 : *(E)-13*-hydroxyoctadec-11-enoic acid

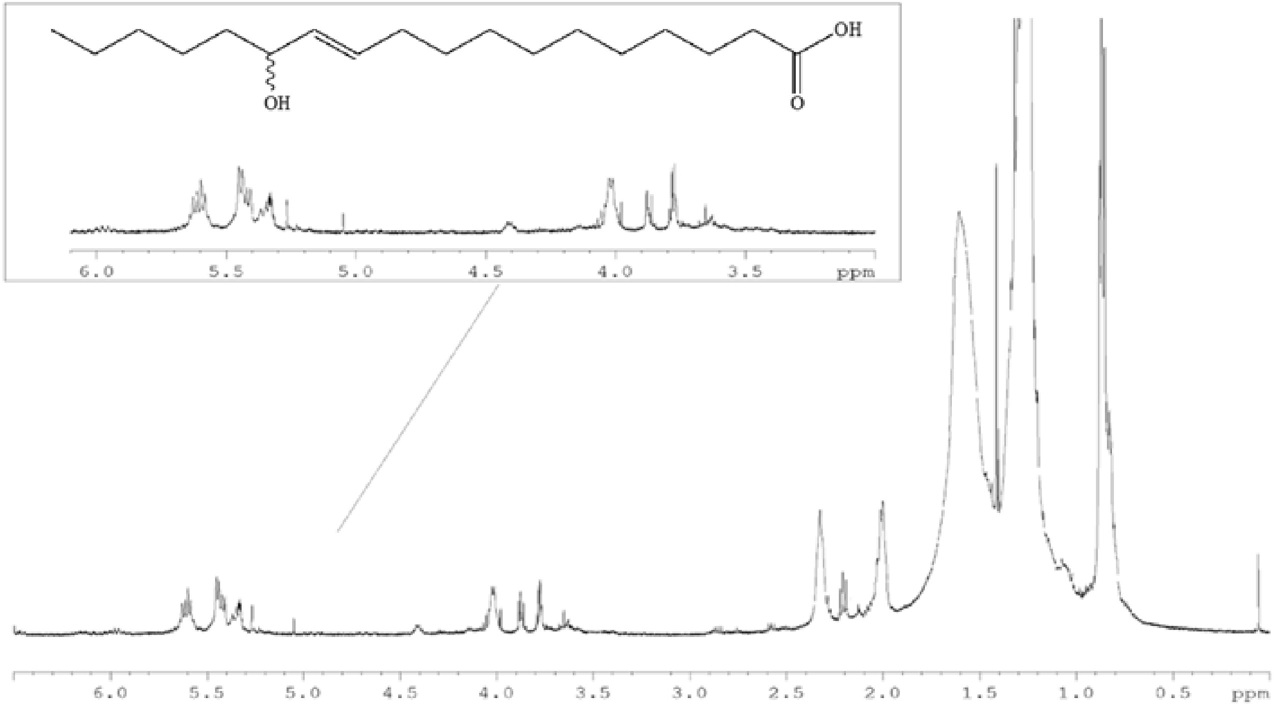

S5. ^{1}H NMR spectrum of compound **2** (500 MHz, $CDCl_3$)

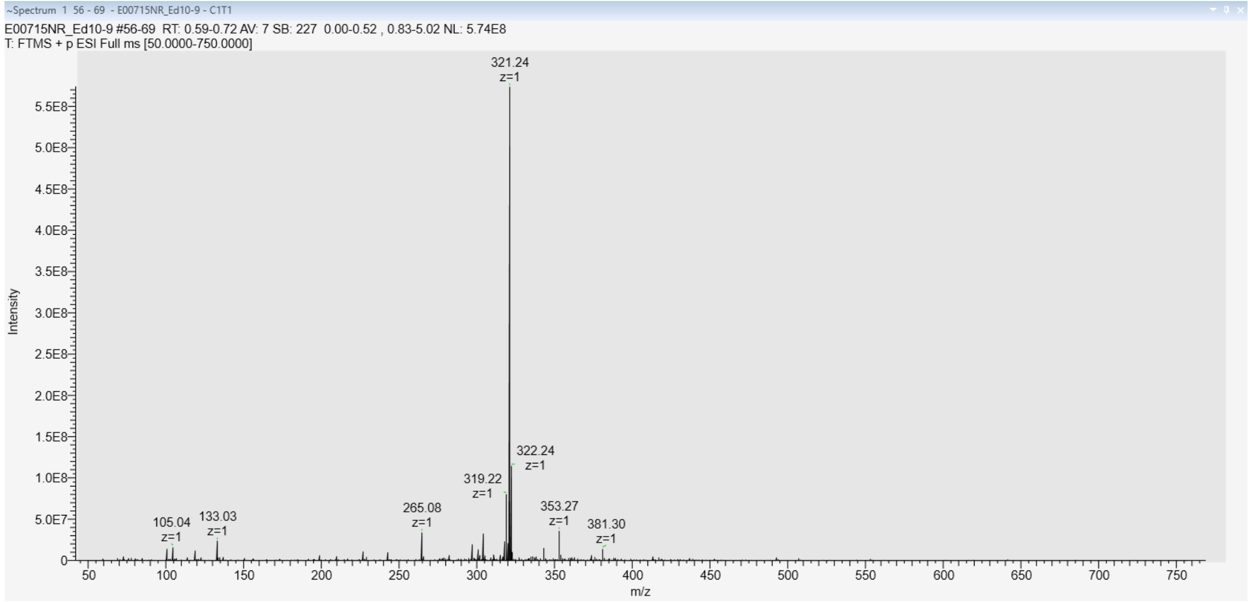

S6. ESIMS spectrum of compound **2**

Compound 3 : (*E*)-heptadeca-1,10-dien-4,6-diyne-3,8,9-triol

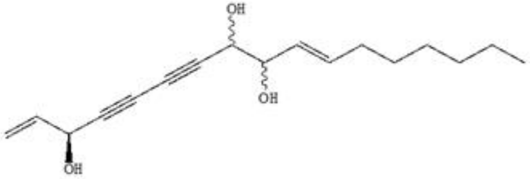

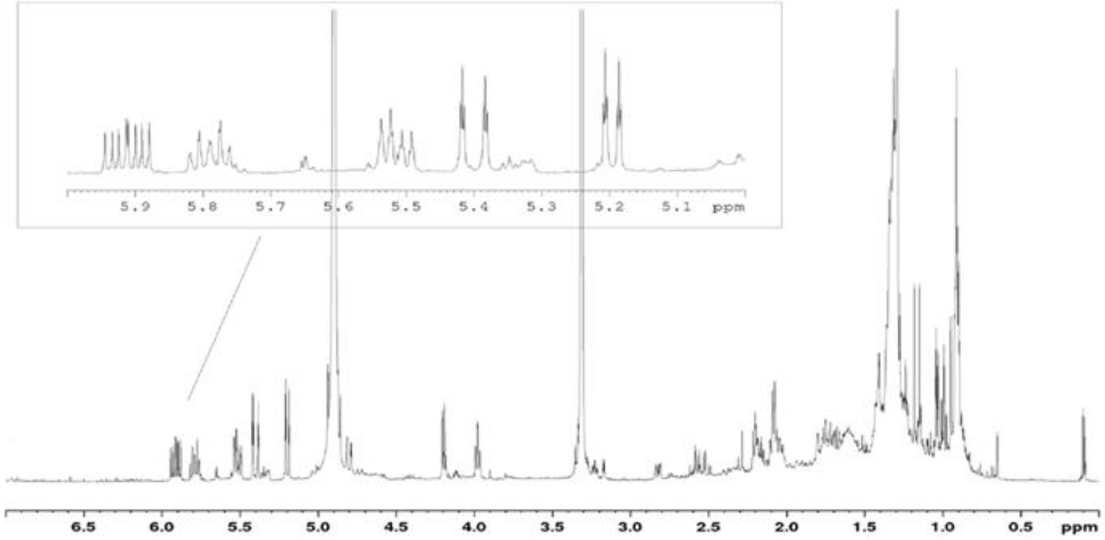

S7. ^{1}HNMR spectrum of compound **3** (500 MHz,CD_3OD)

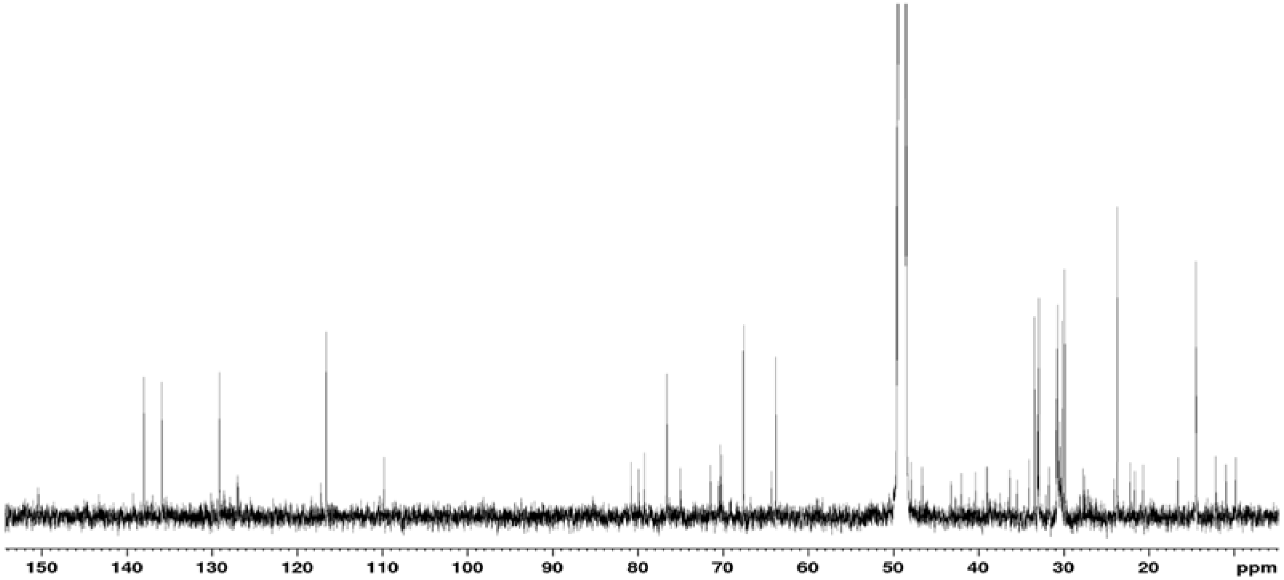

S8. ^{13}C NMR spectrum of compound **3** (125 MHz,CD_3OD)

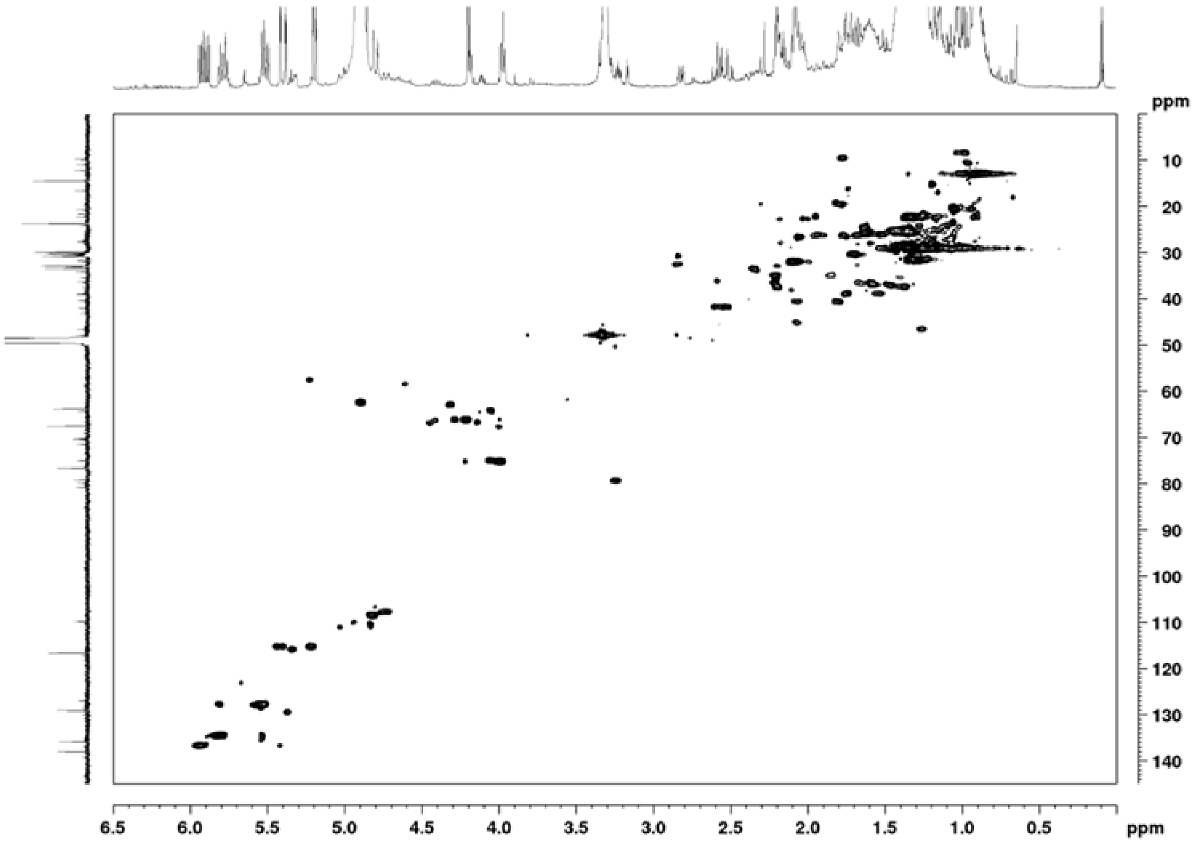

S9. HSQC spectrum of compound **3** (500 MHz,CD_3OD)

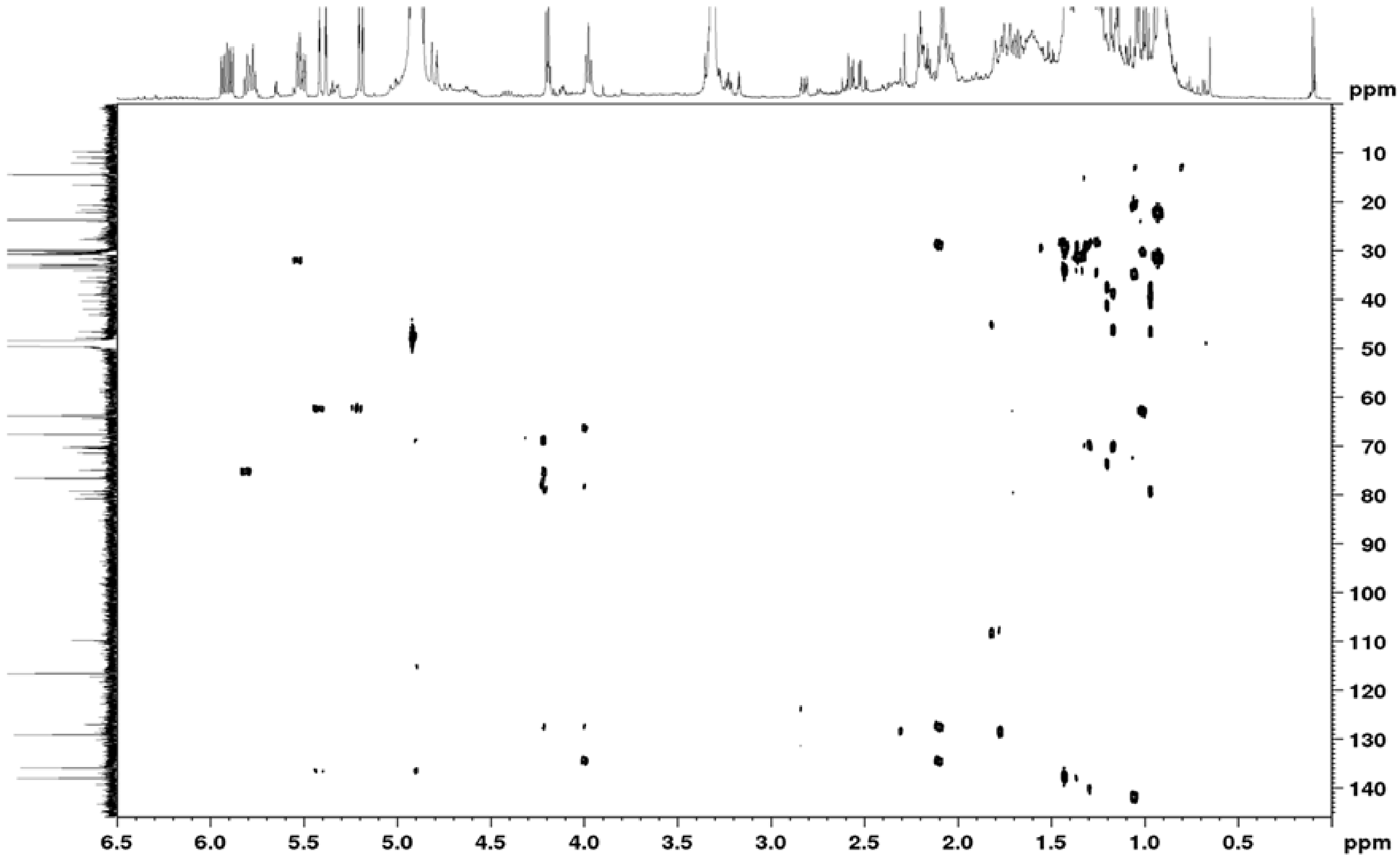

S10. HMBC spectrum of compound **3** (500 MHz,CD_3OD)

Service de Spectrométrie de Masse - Fédération de Chimie Le Bel - UAR2042 - CNRS / UDS

Analysis Info

Analysis Name	O48750NR.d	Acquisition Date	15/12/2025 11:36:19
Method	esi low pos(2025).m	Operator	admin
Sample Name	Ed13-8	Instrument	micrOTOF
Comment			

Acquisition Parameter

Source Type	ESI	Capillary	4500 V	Nebulizer	0.4 Bar	Corona	195 nA
Ion Polarity	Positive	Set Capillary Exit	80.0 V	Dry Gas	4.0 l/min	Set Hexapole RF	70.0 V
n/a	n/a	Set Skimmer 1	50.0 V	Dry Heater	180 °C	APCI Heater	514 °C

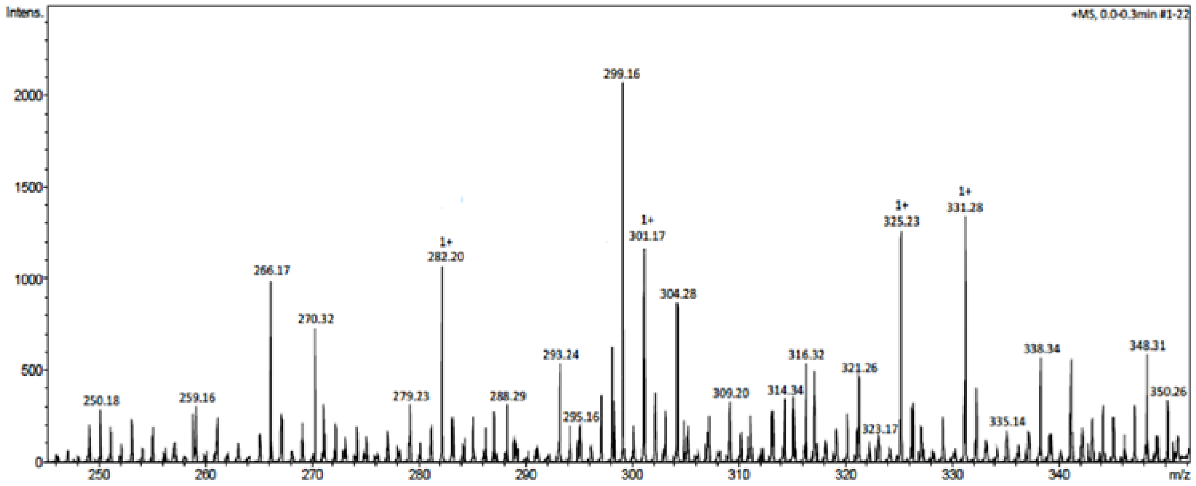

S11. ESIMS spectrum of compound **3**

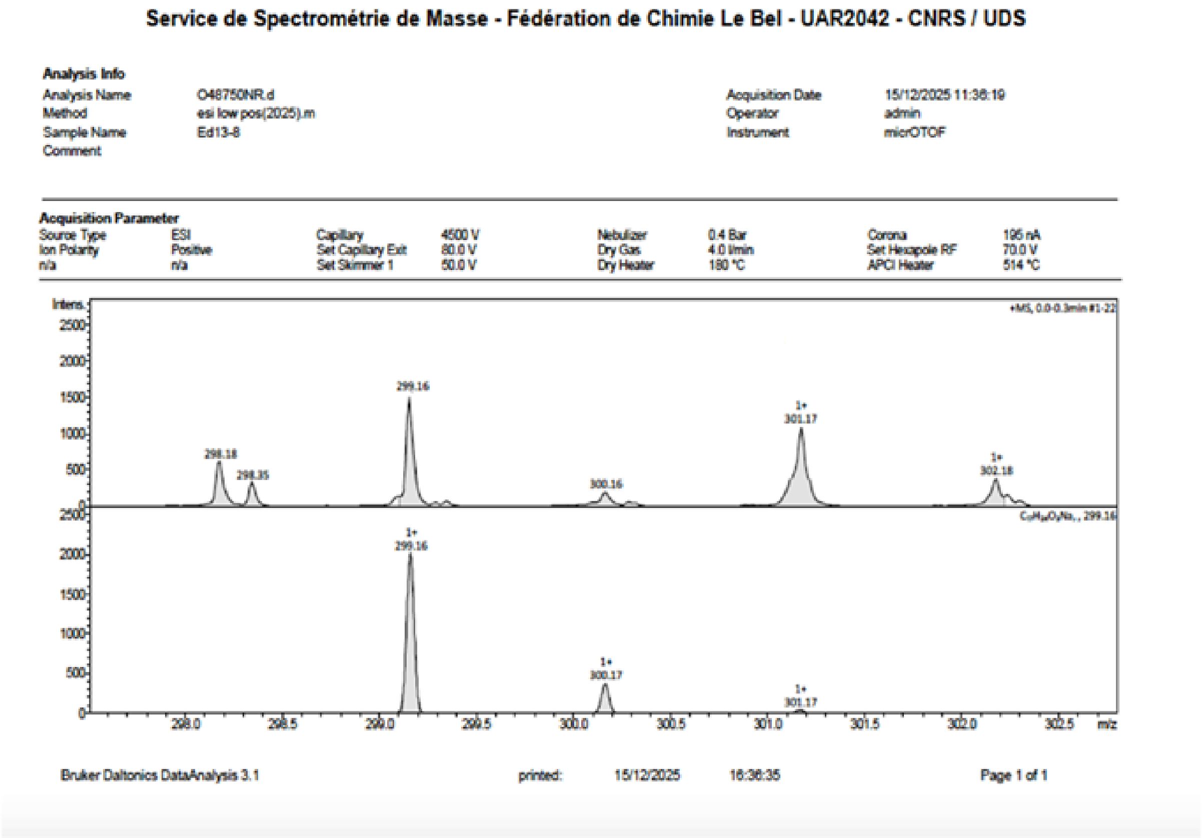

S12. ESIMS spectrum of compound **3**

Compound 4 : (*Z*)-heptadeca-1,9-dien-4,6-diyn-3-ol (Falcarinol)

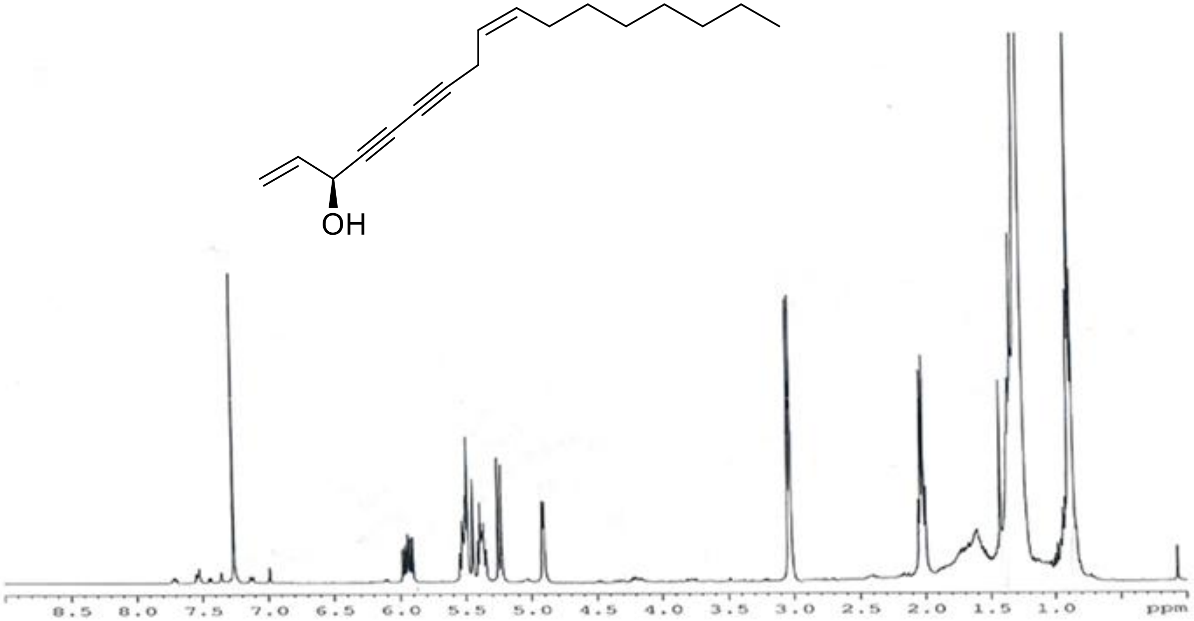

S13. ^{1}H NMR spectrum of compound **4** (500 MHz,CD_3OD)

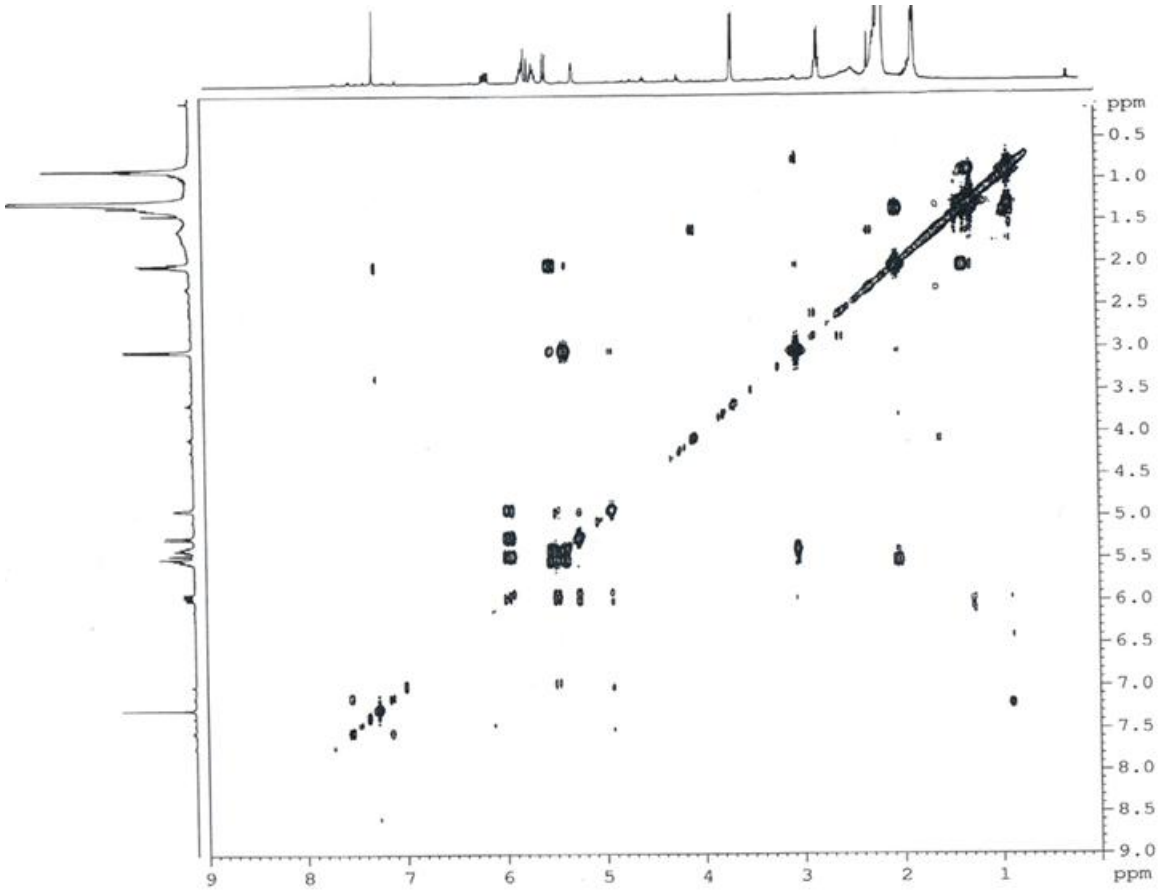

S14. COSY spectrum of compound **4** (500 MHz,CD_3OD)

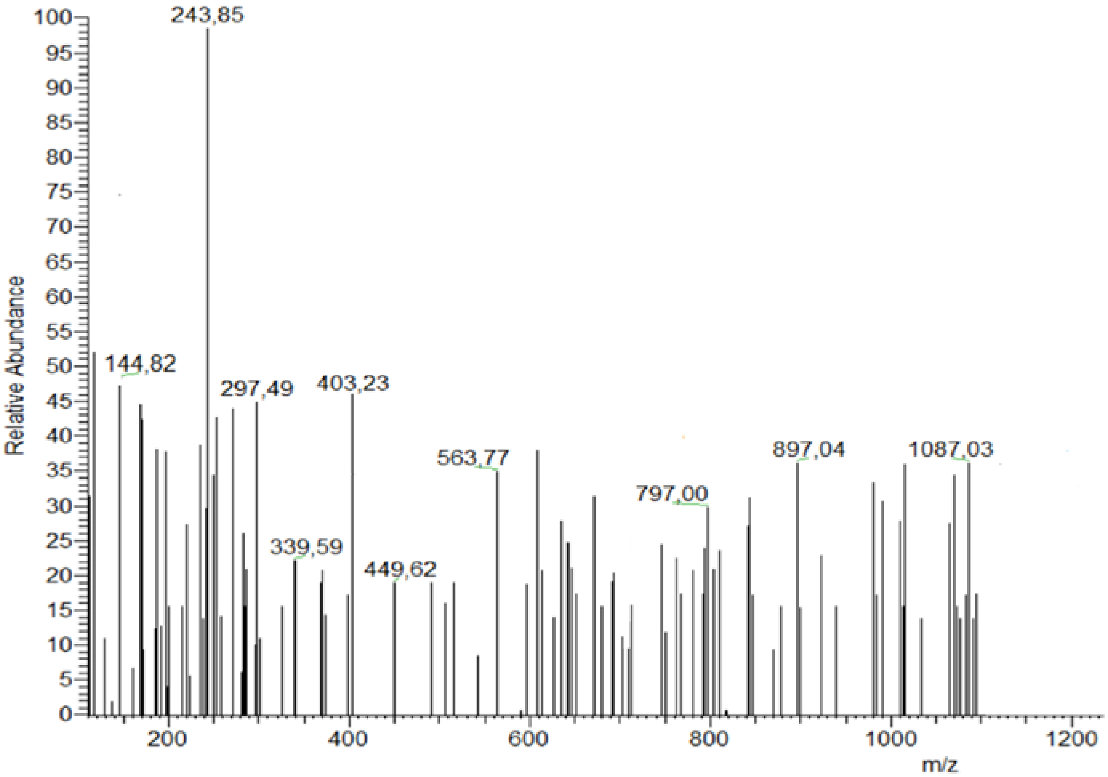

S15. ESIMS spectrum of compound **4**

Compound 5 : Glycerol monopalmitate

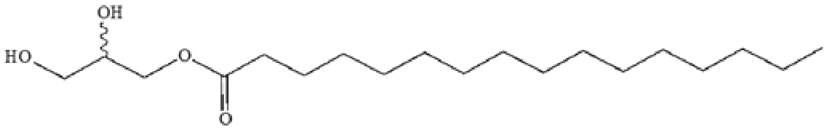

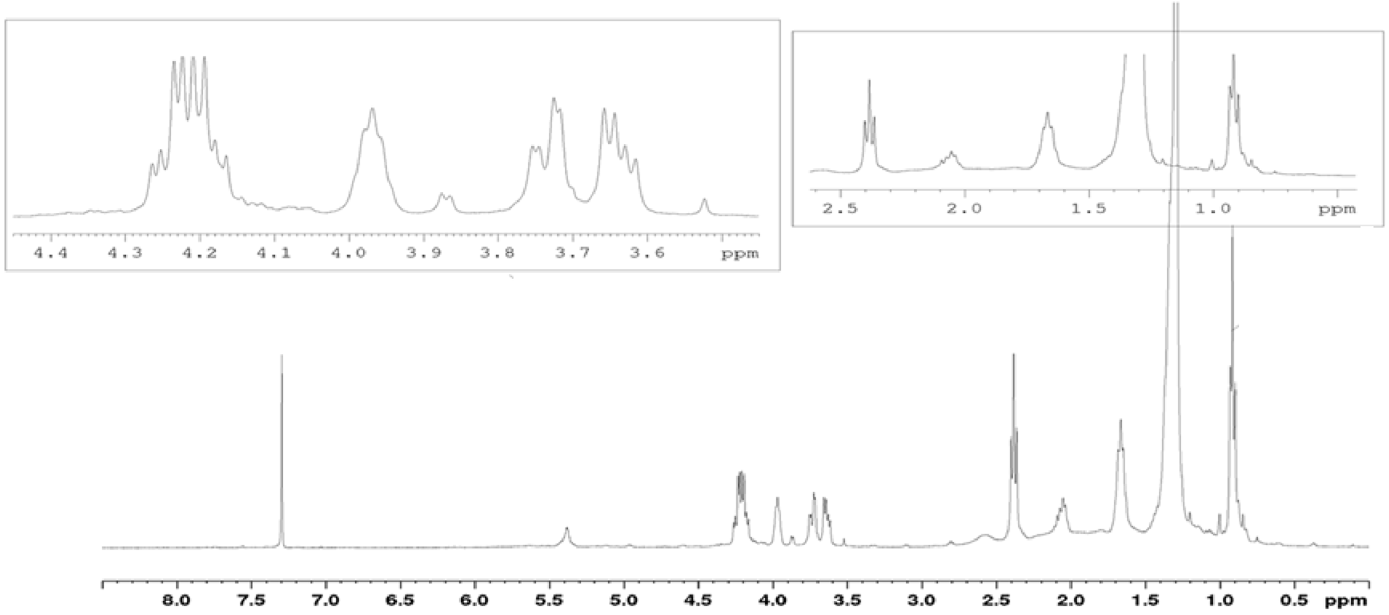

S16. 1H NMR spectrum of compound **5** (500 MHz,CD_3OD)

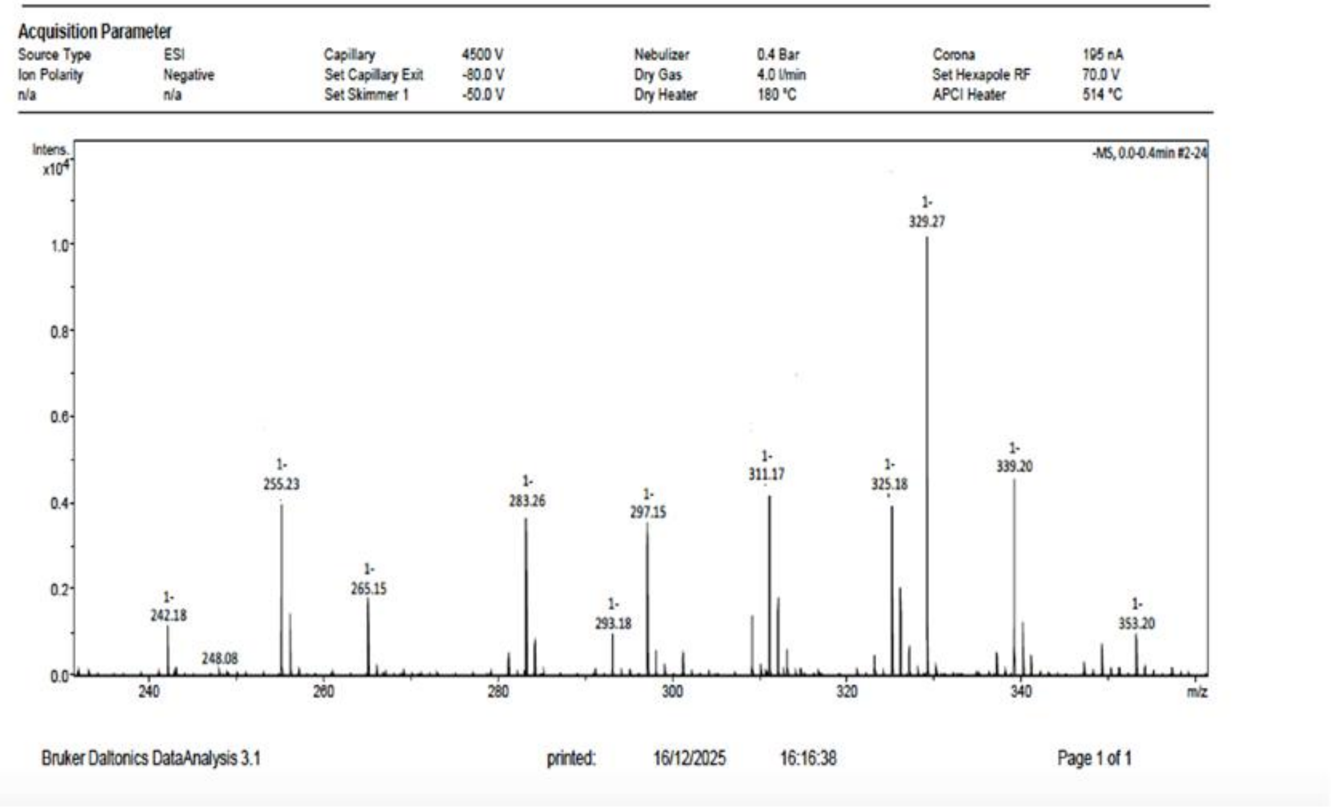

S17. ESIMS spectrum of compound **5**

Acquisition Parameter

Source Type	ESI	Capillary	4500 V	Nebulizer	0.4 Bar	Corona	195 nA
Ion Polarity	Positive	Set Capillary Exit	80.0 V	Dry Gas	4.0 l/min	Set Hexapole RF	70.0 V
n/a	n/a	Set Skimmer 1	50.0 V	Dry Heater	180 °C	APCI Heater	514 °C

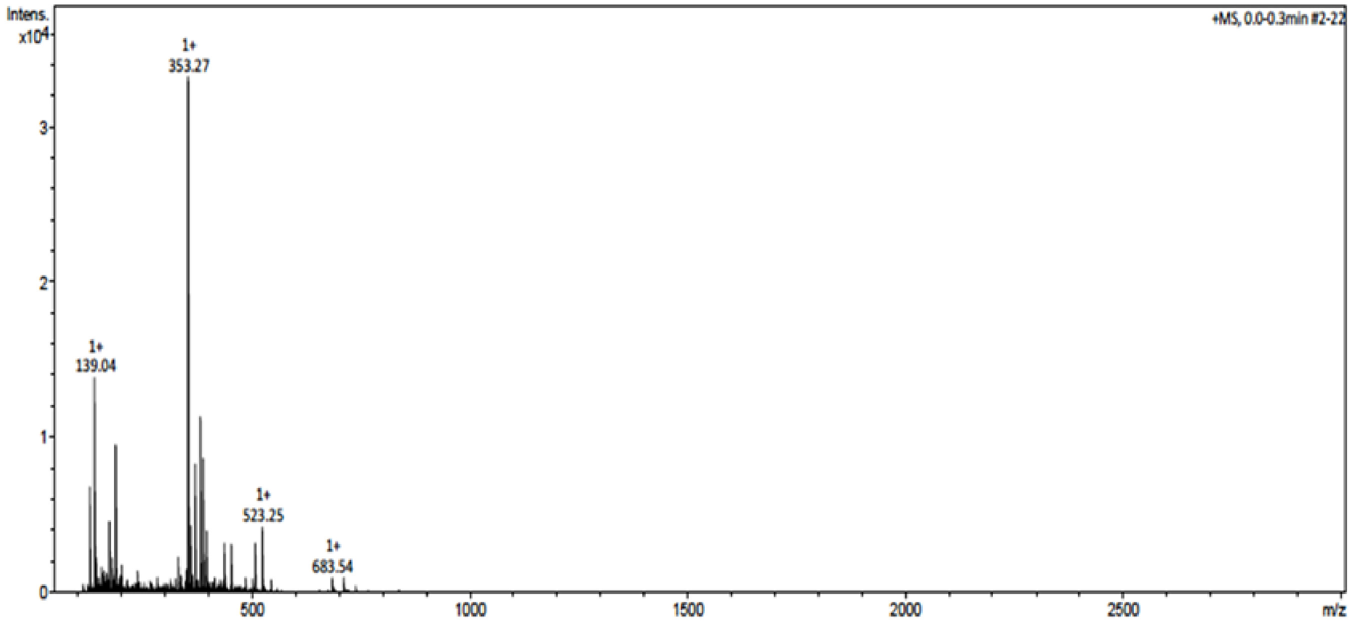

S18. ESIMS spectrum of compound **5**

Compound 6: Kaempferol 3-*O*-β-D-glucopyranosyl-(1→6)-*O*-β-D-galactopyranoside

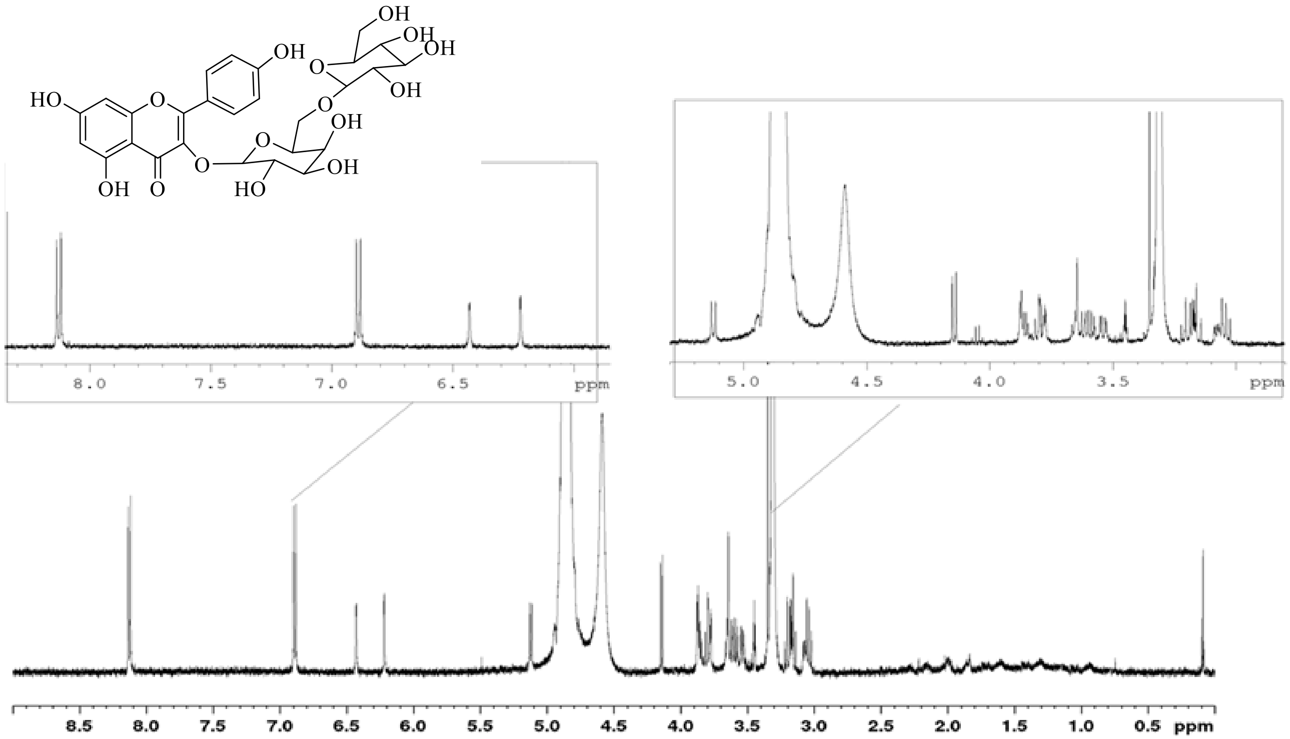

S19. ^{1}H NMR spectrum of compound **6** (500 MHz,CD_3OD)

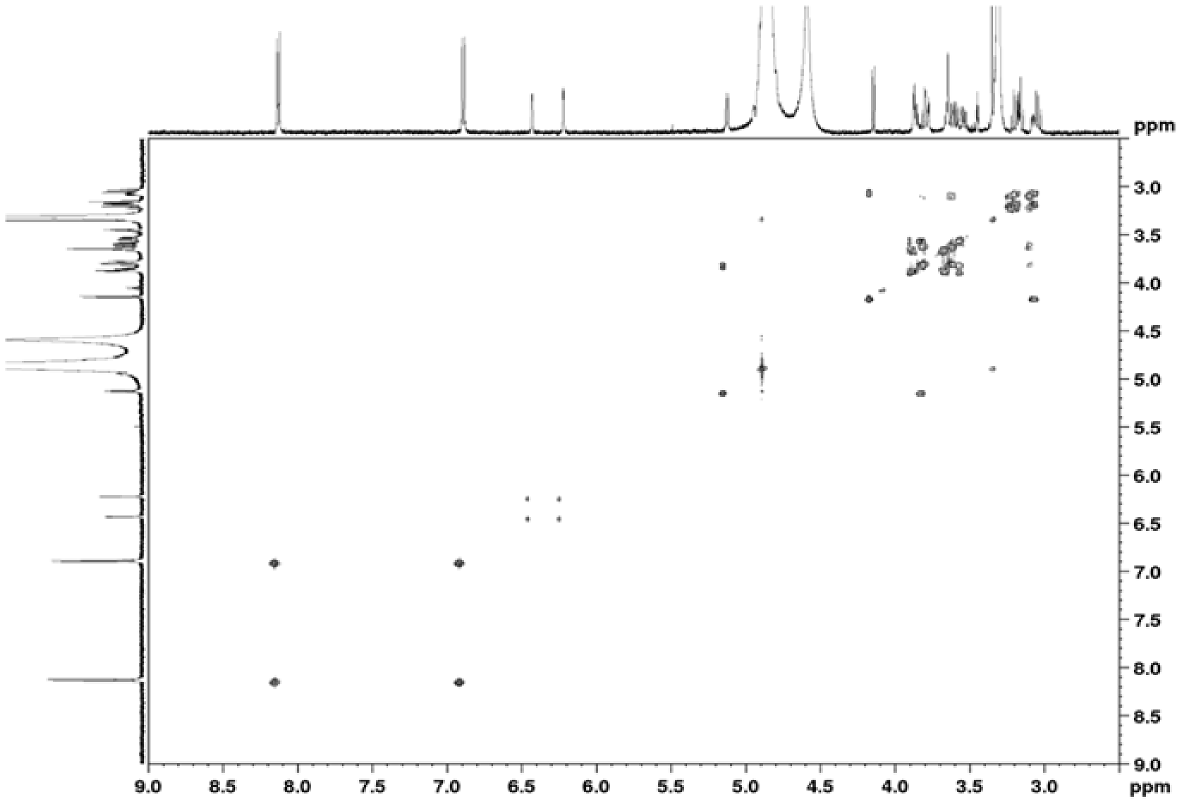

S20. COSY spectrum of compound **6** (500 MHz, CD_3OD)

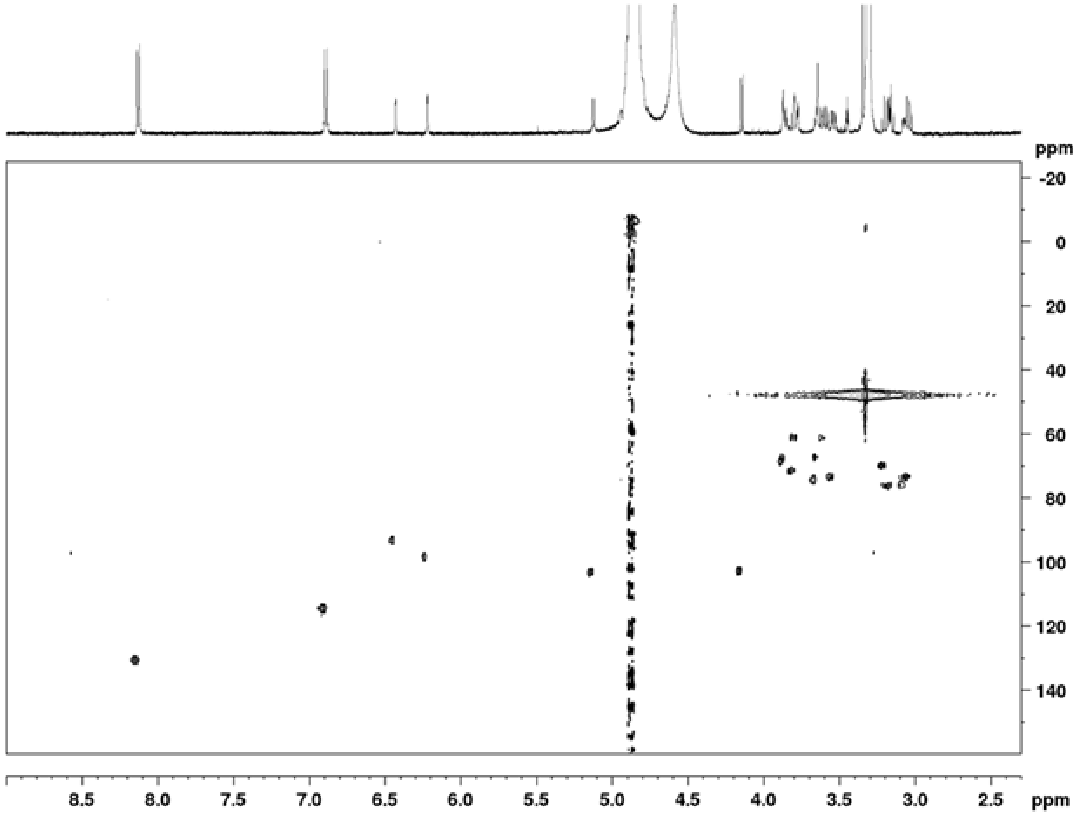

S21. HSQC spectrum of compound **6** (500 MHz,CD_3OD)

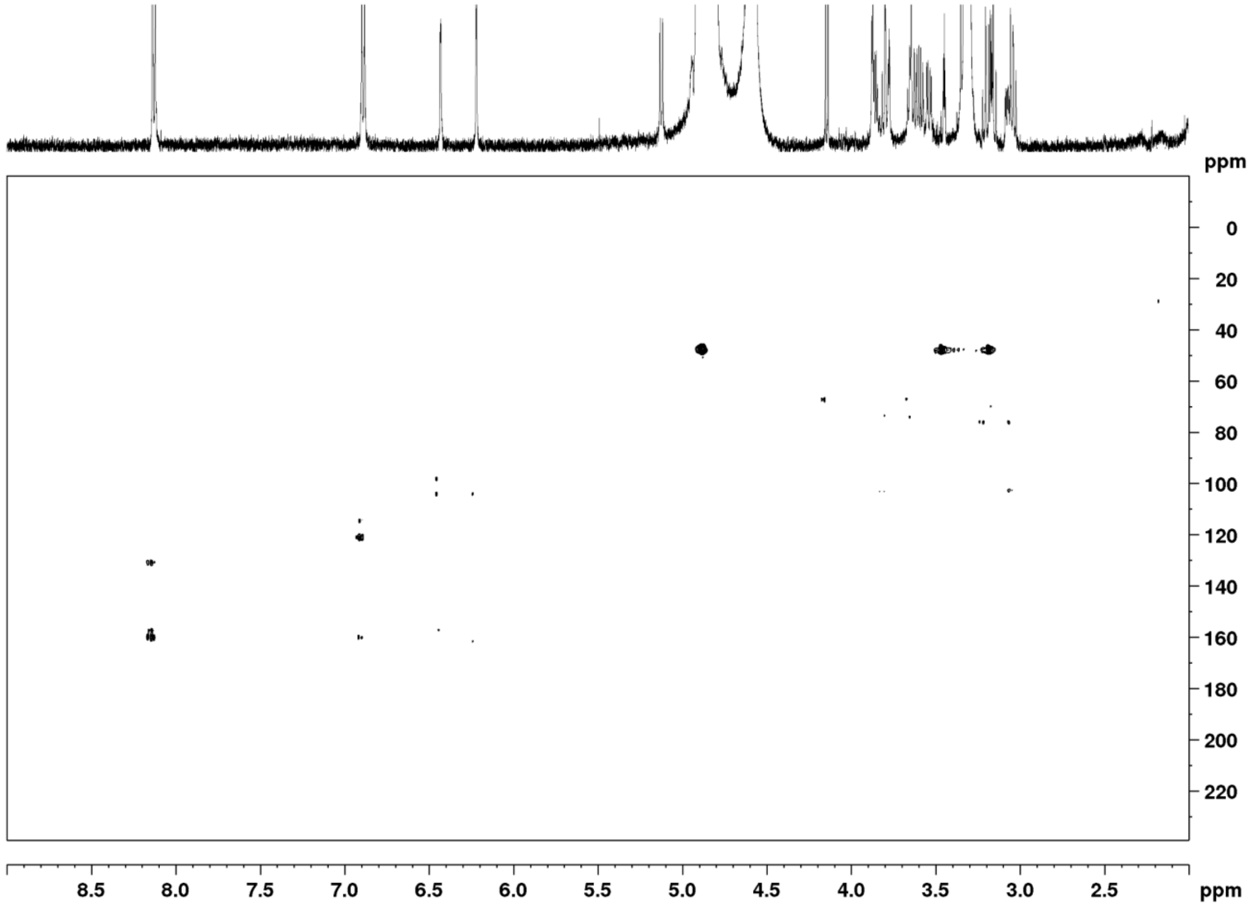

S22. HMBC spectrum of compound **6** (500 MHz, CD_3OD)

Service de Spectrometrie de Masse - Federation de Chimie Le Bel - UAR 2042 - CNRS / Unistra

Analysis Info

Analysis Name	F17541SK.d	Acquisition Date	10/8/2024 2:39:59 PM
Method	Tune_pos_Mid.m	Operator	BDAL@DE
Sample Name	Edb2-4 NEG	Instrument	micrOTOF II

Acquisition Parameter

Source Type	ESI	Capillary	3000 V	Nebulizer	0.4 Bar	Set Hexapole RF	330.0 Vpp
Ion Polarity	Negative	Dry Heater	200 °C	Dry Gas	4.0 l/min	Set Capillary Exit	-150.0 V

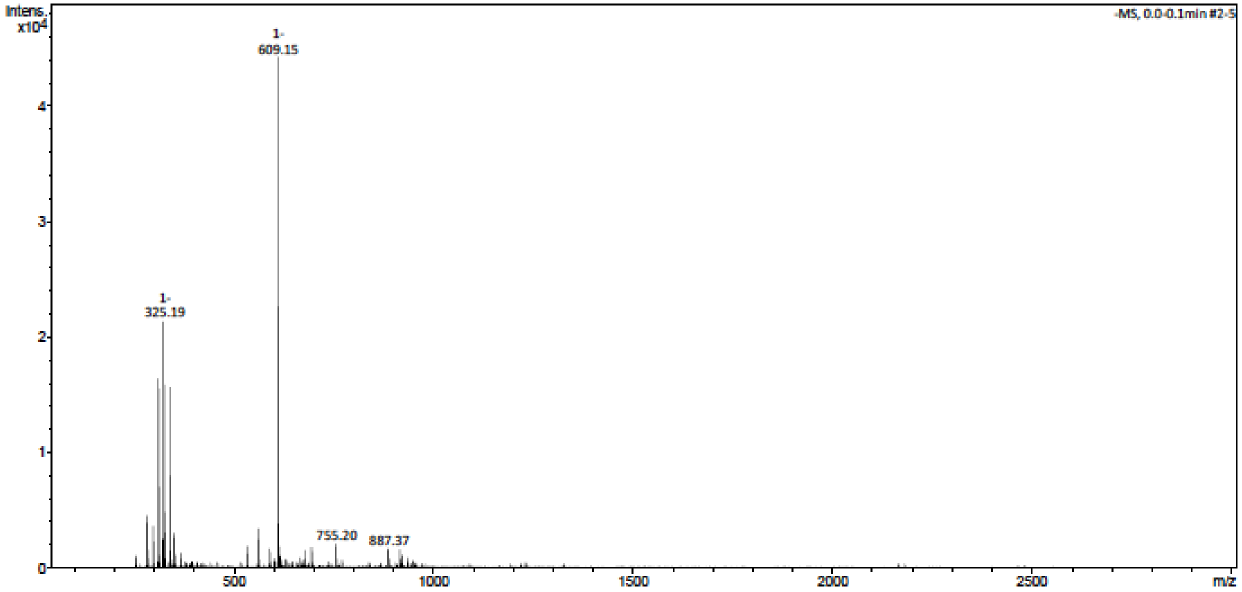

S23. ESIMS spectrum of compound **6**

Service de Spectrometrie de Masse - Federation de Chimie Le Bel - UAR 2042 - CNRS / Unistra

Analysis Info

Analysis Name	F17540SK.d	Acquisition Date	10/8/2024 2:38:20 PM
Method	Tune_pos_Mid.m	Operator	BDAL@DE
Sample Name	Edb2-4	Instrument	micrOTOF II

Acquisition Parameter

Source Type	ESI	Capillary	4500 V	Nebulizer	0.4 Bar	Set Hexapole RF	330.0 Vpp
Ion Polarity	Positive	Dry Heater	200 °C	Dry Gas	4.0 l/min	Set Capillary Exit	150.0 V

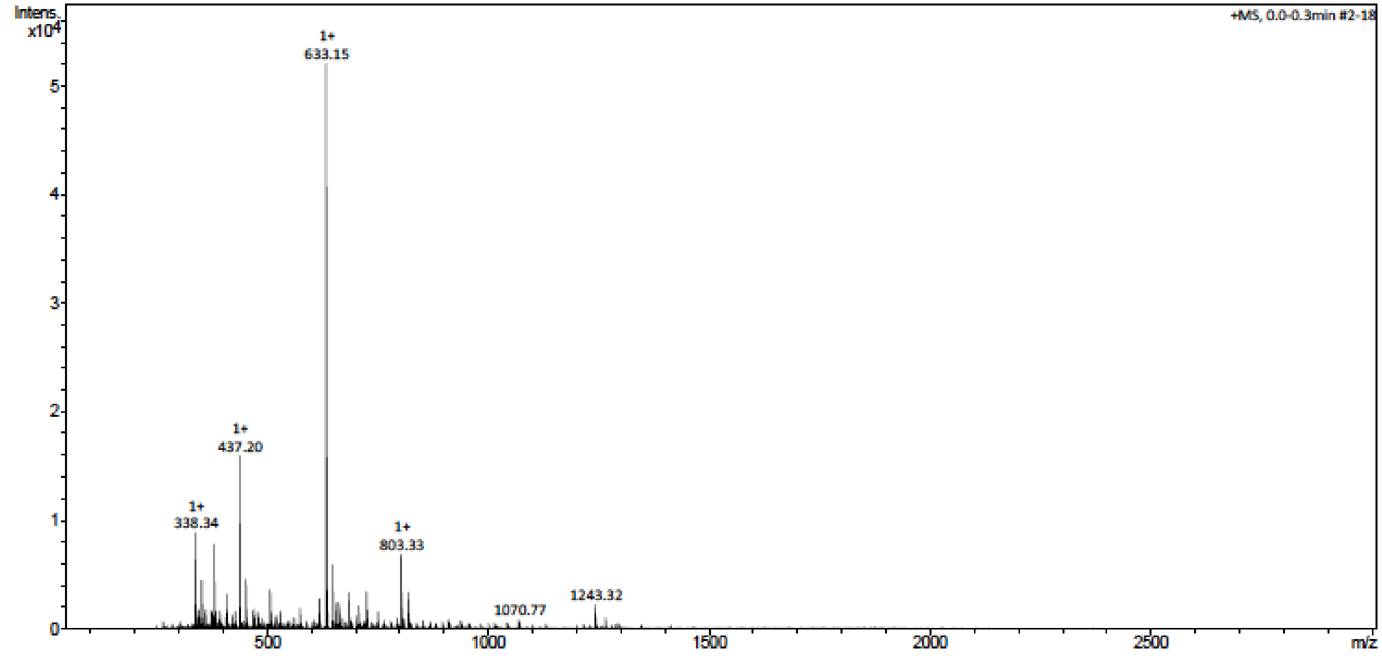

S24. ESIMS spectrum of compound **6**

Compound 7 : Quercetin 3-*O*-β-D-glucopyranosyl-(1→6)-*O*-β-D-galactopyranoside

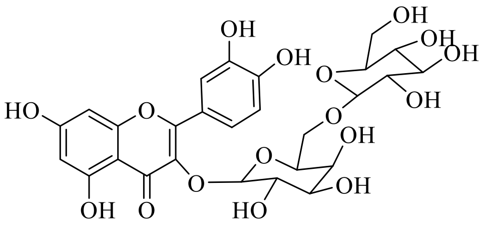

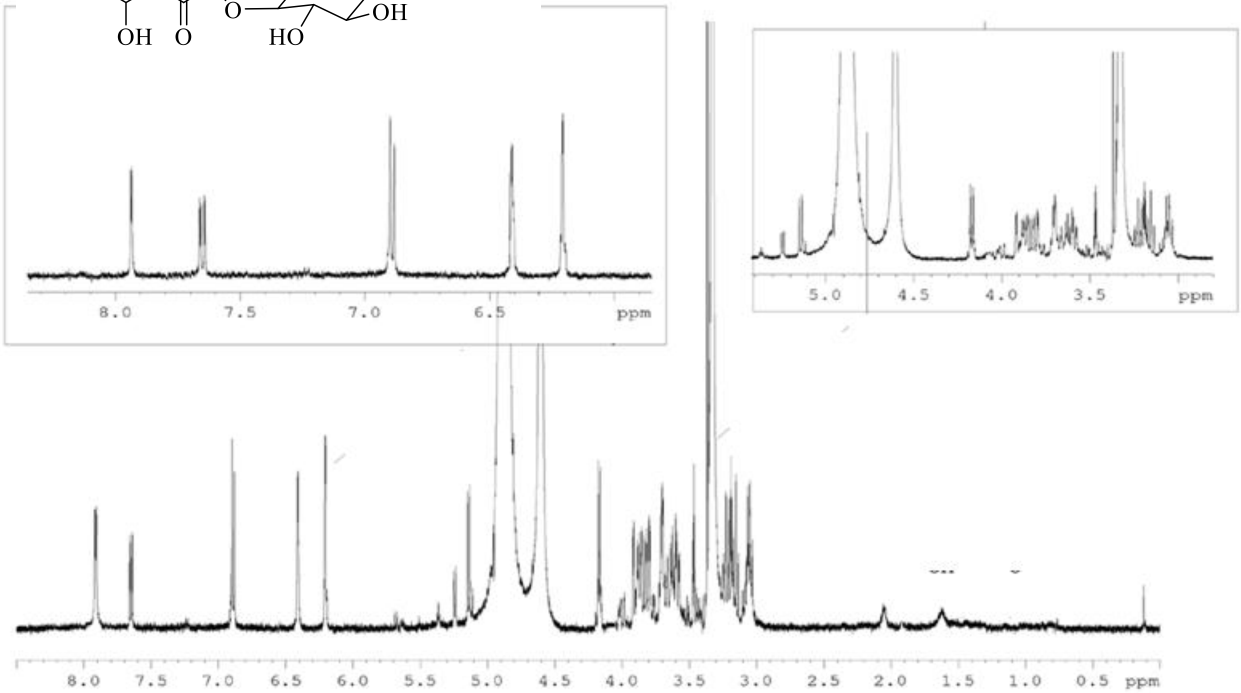

S25. ^{1}H NMR spectrum of compound **6** (500 MHz,CD_3OD)

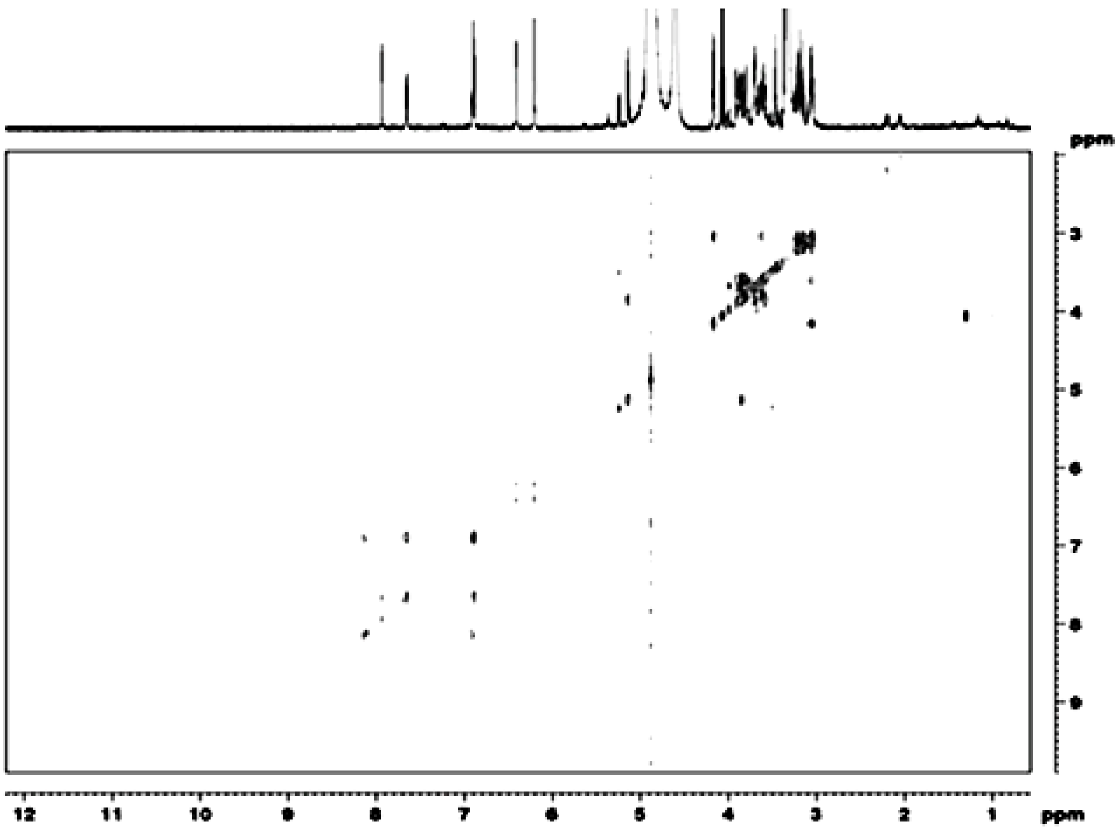

S26. COSY(H,H) spectrum of compound **7** (500 MHz,CD_3OD)

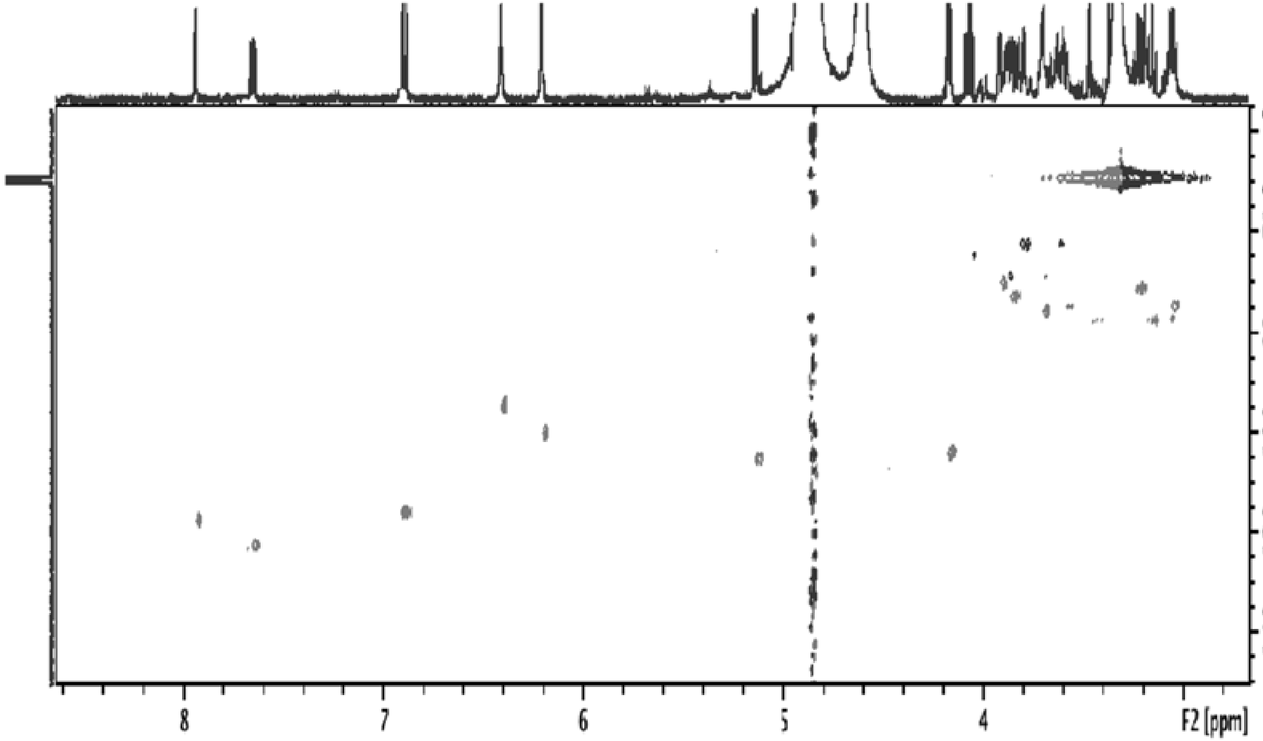

S27. HSQC spectrum of compound **7** (500 MHz,CD_3OD)

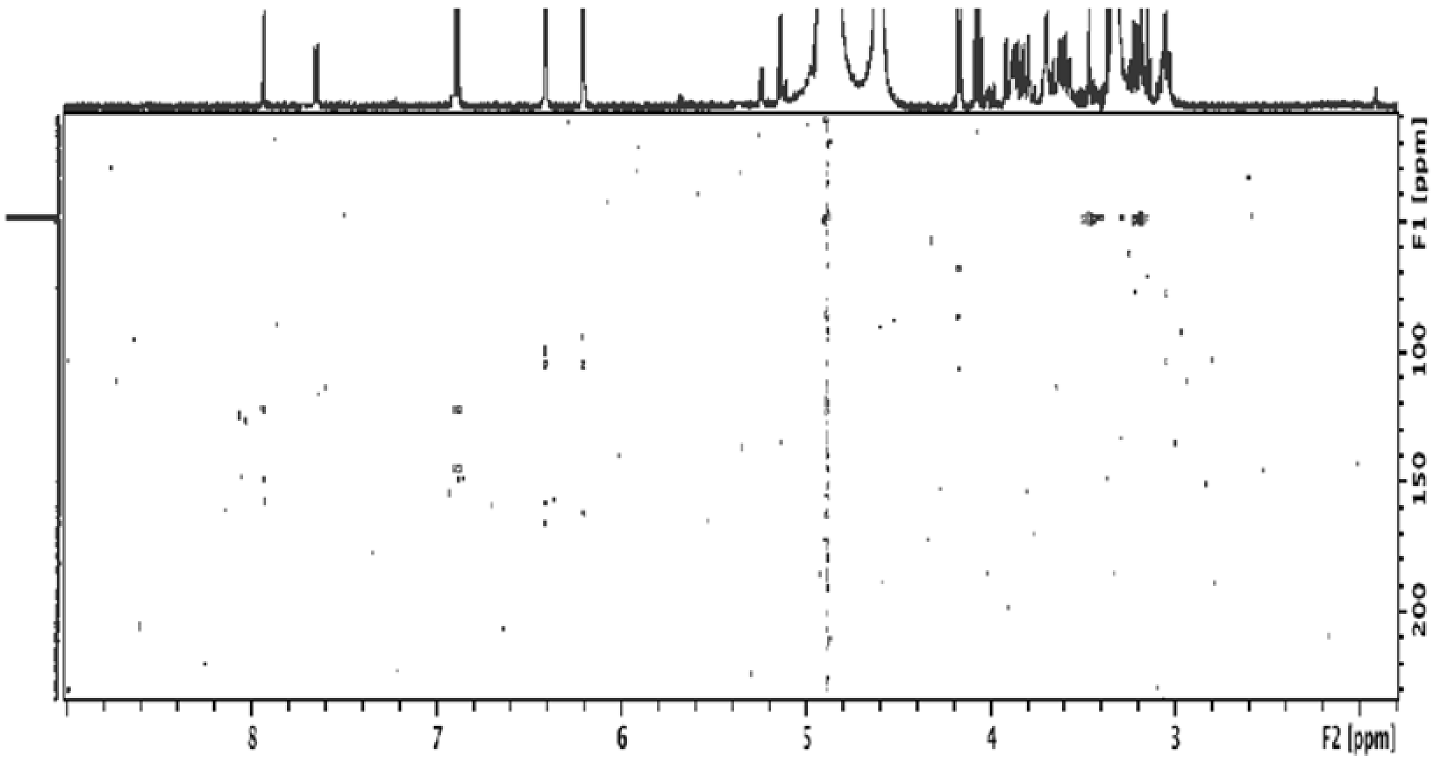

S28. HMBC spectrum of compound **7** (500 MHz,CD_3OD)

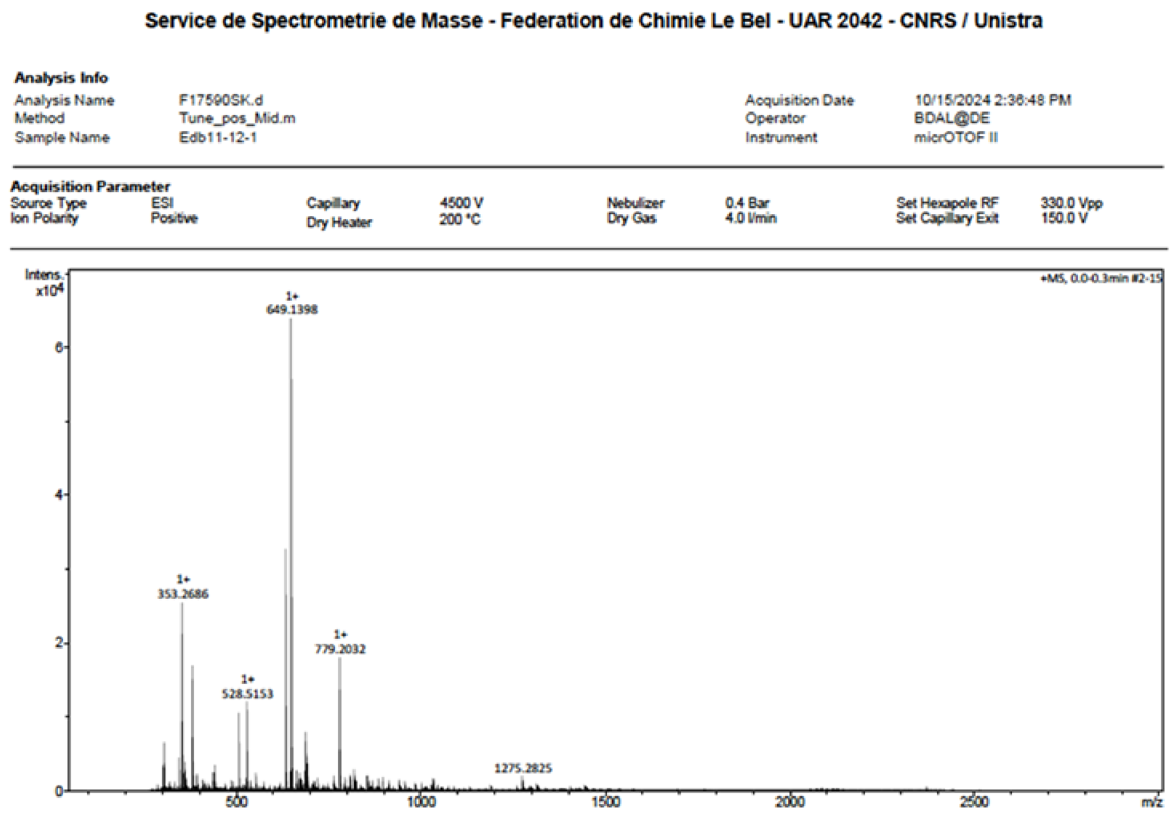

S29. ESIMS spectrum of compound **7**

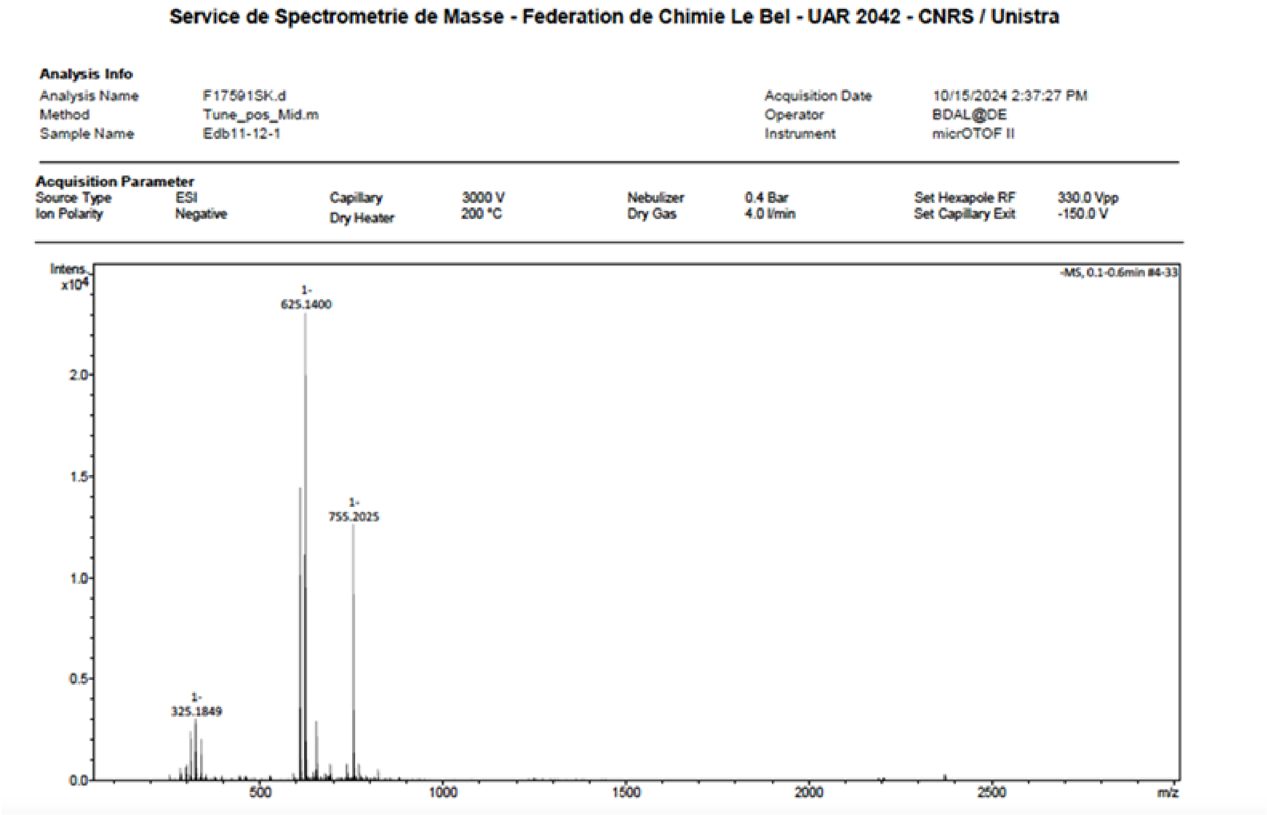

S30. ESIMS spectrum of compound **7**
